# Candidacidal effect of *Moringa* stabilized silver nanomaterials reveal disruption of cell wall integrity, efflux pump, vacuole homeostasis and virulence traits in *Candida auris*

**DOI:** 10.1371/journal.pone.0336309

**Published:** 2025-11-19

**Authors:** Insha Nahvi, Suriya Rehman, Ankush Kaushik, B. Rabindran Jermy, Sultan Akhtar, Mohd Farhan, Zeeshan Fatima, Saif Hameed

**Affiliations:** 1 Amity Institute of Biotechnology, Amity University Haryana, Manesar, Gurugram, India; 2 Department of Epidemic Diseases Research, Institute for Research and Medical Consultations (IRMC), Imam Abdulrahman Bin Faisal University, Dammam, Saudi Arabia; 3 Department of Nanomedicine Research, Institute for Research and Medical Consultations (IRMC), Imam Abdulrahman Bin Faisal University, Dammam, Saudi Arabia; 4 Department of Biophysics, Institute for Research and Medical Consultations (IRMC), Imam Abdulrahman Bin Faisal University, Dammam, Saudi Arabia; 5 Department of Chemistry, College of Science, King Faisal University, Al Ahsa, Saudi Arabia; Cleveland Clinic Lerner Research Institute, UNITED STATES OF AMERICA

## Abstract

*Candida auris* (*C. auris*) is known as a superbug characterized by its high prevalence of multi-drug resistance, causing outbreaks in healthcare settings and leading to high mortality rates worldwide. Developing novel and efficient measures to combat its spread and mitigate its impact on patient outcomes is pertinent. This study describes a simple and eco-friendly method for synthesizing silver nanocomposites and silver-zinc nanocomposites using leaf extract of the *Moringa Oleifera (MO),* plant. The synthesized nanocomposite was characterized and confirmed by several techniques like Fourier Transform Infrared Spectroscopy (FTIR), X-ray diffractometry (XRD), Thermogravimetric Analysis (TGA), X-ray Photoelectron Spectroscopy (XPS), Scanning electron microscopy (SEM). Moreover, nanocomposites were observed to display effective action against *C. auris.* Minimum inhibitory concentration (MIC) of Ag-*MO* and Ag-Zn-*MO* nanocomposite was found to be 125 µg/mL and 250 µg/mL respectively. Ag-*MO* was found to be fungicidal in nature while Ag-Zn-*MO* was fungistatic at their respective MIC’s. Mechanistic insights elucidate changed cell wall composition, impaired efflux activity, dysfunctional vacuole and suppressed biofilm formation. *In vivo* efficacy was demonstrated *via* nematode model survival and macrophage mediated killing. Both the nanocomposites enhanced *Caenorhabditis elegans* survival which was 45% in Ag-*MO*, 41% in Ag-Zn-*MO* vs. 30% in untreated and also enhanced macrophage mediated killing of *C. auris*. The current study highlights the potential application of *Moringa* synthesized AgNPs and Ag-ZnNPs as promising approach to combat *C. auris* infections in particular post its biocompatibility studies.

## 1. Introduction

*C. auris* is a significant global health concern, causing a variety of infections including vaginitis, oral thrush, skin infections, bloodstream infections, and systemic diseases. These opportunistic pathogens are responsible for a substantial number of deaths worldwide [1]. Although the exact pathophysiology of *Candida* infections is unknown, their prevalence is rising rapidly. The increasing resistance to traditional antifungal drugs has highlighted the urgent need for effective prevention and early diagnosis. Fungal infections pose a significant and escalating danger to global health, trailing only viruses and bacteria in their threat level. A major concern is the increasing resistance of fungi to existing antifungal medications, making treatment more challenging. Developing new antifungal drugs is inherently difficult due to the eukaryotic nature of fungal cells, which are similar to human cells, limiting drug targets. Compounding this issue, rising global temperatures and increased humidity create ideal conditions for fungal proliferation, potentially leading to widespread outbreaks or even pandemics. The world is also seeing a continuous rise in the number of immunocompromised individuals, whose weakened immune systems make them particularly vulnerable to severe fungal infections. This confluence of factors is pushing pharmaceutical companies to urgently seek innovative strategies to combat the growing global burden of fungal diseases.

Azoles are the most used antifungal agents for *Candida* infections, but the emergence of drug resistance has become a rising obstacle against efficient therapeutics [2]. The inconsistent response of *C. auris* to various antifungal treatments, alongside high fluconazole resistance, results in widely fluctuating mortality rates (0–72%), highlighting the unpredictability of infection outcomes. In response to these persistent issues, modern research is increasingly focused on nanoparticles, using them as carriers or adjuvants to boost the performance of current medications [2–4].

Nanotechnology offers promising solutions to address the challenges associated with *Candida* infections. Metal nanoparticles, particularly noble metals like gold, silver, and platinum, have garnered attention due to their unique optical, electrical, and antimicrobial properties [4,5]. These nanoparticles have potential applications as antifungal agents, offering an alternative to traditional treatments [6]. While physical methods for nanoparticle synthesis are preferred to minimize toxic waste, there is growing interest in developing eco-friendly wet-synthesis approaches using natural products [7,8]. These methods aim to provide efficient, cost-effective, and sustainable ways to produce high-quality metal nanoparticles for medical applications. When metal nanoparticles are being synthesized, phytomolecules, which are natural compounds extracted from plants, fungi, and bacteria work well as reducing agents. These compounds, with their diverse chemical structures, help convert metal ions into nanoparticles and stabilize the resulting particles [8]. Nanomaterials synthesized using plant extracts have found numerous applications in the biomedical field, including antimicrobial and antifungal properties, biosensing, and other biological uses [9]. Biologically synthesized silver nanoparticles have been extensively utilized in various applications, including antimicrobial substances, coatings, biosensors, and catalysts. They also exhibit biological properties like anti-platelet and anti-HIV activities. The potential of metal-based nanoparticles against fungal infections is evident from a wide range of studies. According to a study by Kaplan et al, biosynthesized silver nanoparticles exhibited a potent antifungal activity against *C. albicans* and *C. utilis* [10].

The plant *Moringa oleifera* is indigenous to South America, Asia, and Africa and has long been recognized by traditional healers for its medicinal properties, particularly in treating inflammation and bacterial infections [11]. Numerous studies have highlighted *MO*’s diverse health benefits, such as diuretic, cardiovascular, hepatoprotective, hypoglycemic, immunomodulatory, anti-inflammatory, anti-cancer, antiulcer, antihypertensive, antidiabetic, analgesic, anti-aging, and cardiovascular properties. The plant’s hypotensive, antibacterial, and anticancer effects are attributed to specific chemical compounds found in its leaves and fruits. Various methods have been developed to extract the bioactive compounds from *MO* for use in food supplements and medicines, and to further explore its health benefits. The diverse phytochemical profile of *Moringa* species, encompassing alkaloids, saponins, tannins, steroids, phenolic acids, glucosinolates, flavonoids, and terpenes, contributes to their pharmacological potential [12].

In this study, silver nanocomposite (Ag-*MO*s) and bimetallic nanocomposite, silver-zinc nanocomposite (Ag-Zn-*MO*) were synthesized using the *MO,* plant as a reducing agent. Our results indicated that these nanocomposites have a promising and potential antifungal effect. The synthesized nanomaterial was characterized using Fourier-transform infrared spectroscopy (FT-IR), X-ray diffraction spectroscopy (XRD), X-ray photoelectron spectroscopy (XPS), Zeta potential analysis, and scanning electron microscopy (SEM). Additionally, during this study, the antifungal activity of the green-synthesized Ag-*MO* nanocomposites and Ag-Zn-*MO* nanocomposites against *C. auris* along with mechanisms of action were also evaluated.

## 2. Materials and methods

### 2.1. Reagents/abbreviation

Agar, nutritional broth, YPD (Yeast Extract Peptone Dextrose), YNB (Yeast Nitrogen Base) devoid of amino acids and ammonium sulfate, Propidium iodide (PI), n-heptane, rhodamine 6G (R6G), 2-deoxyglucose (2-DOG), 2,4-dinitrophenol (2,4-DNP), and Brain Heart Infusion (BHI) were acquired from Himedia (Mumbai, India). Fisher Scientific provided the following: sodium chloride (NaCl), mannitol, potassium chloride (KCl), di-sodium hydrogen orthophosphate (Na₂HPO₄), dipotassium hydrogen orthophosphate (K₂HPO₄), potassium dihydrogen orthophosphate (KH₂PO₄), sodium hydroxide (NaOH), potassium hydroxide (KOH), D-glucose, dimethyl sulfoxide (DMSO), sodium dodecyl sulfate (SDS), ammonium sulfate (NH₄)₂SO₄, ammonium chloride (NH₄Cl), and Triton-X. 3-[4, 5-dimethylthiazol-2-yl] tetrazolium salt Trypan blue and −2, 5-diphenyltetrazolium bromide (MTT) were acquired from SRL, Mumbai. Purchased from Sigma Chemical Co. (St. Louis, MO, USA) are Calcofluor White (CFW), Congo Red (CR), Fluconazole (FLC), Caspofungin (CAS), Amphotericin B (AMP B), Rhodamine 123, DCFDA (2,7-dichlorofluorescein diacetate), DAPI (4′,6-diamidino-2-phenylindole), and Aniline Blue (AB). Thermo Fisher Scientific, Invitrogen, and the USA are suppliers of FM4–64 dye. Purchased quinacrine dihydrochloride from Merck, Germany.

**Ethical Approval**

Not applicable

### 2.2. Preparation of nanocomposites

Green and healthy leaves of *MO* from Dammam, Saudi Arabia were collected, washed and sun dried. Dried leaves were ground into powder form and sealed into labelled bags until used. For the biosynthesis of nanocomposites, this powder was dissolved in de-ionized water in a 250 mL of Erlenmeyer flask. After boiling with stirring, the solution mixture was filtered to obtain a clear solution and stored at 4 °C. Subsequently, 1 mM of silver nitrate solution that had been prepared at 80°C in a ratio of silver nitrate solution 80: plant extract 20 was added dropwise, stirred and dried [13,14]. To achieve this, the solution was put at room temperature overnight on a shaker for agitation under observation, until the appearance of color changes from light yellow to dark brown. In the case of bimetal silver-zinc nanoparticle mixture, zinc acetate was added along with AgNO_3_ solution, and the similar procedure was repeated.

### 2.3. Characterization analysis of nanomaterial

The phase analysis of Ag, ZnO and Ag-ZnO NPs were analyzed using benchtop XRD (Miniflex 600, Rigaku, Japan). For sample loading, the cavity of the quartz sample holder was filled from the back with both the nanocomposites one by one and the powder was gently compressed and flattened using a clean, flat glass slide so that its surface was flushed with the holder’s surface (15). The sample scan range was performed between 2–80º 2θ scan range at the scanning rate of 1º/min. The textural features, including the BET surface area, pore volume and pore size, were measured using ASAP-2020 plus (Micromeritics, Norcross, GA, USA) based on the nitrogen adsorption technique. The silver nanoparticle chemical coordination was analyzed using DRS-UV-visible spectroscopy analysis (JASCO, Tokyo, Japan). The thermogravimetric analysis of Ag, ZnO and Ag-ZnO NPs derived from *Moringa oleifera* leaves ZIF-8, ZIF-8/ were measured using TGA-DTA (STA 6000, Perkin Elmer, USA). About 20 mg of sample was loaded by placing into the tared pan of the instrument. And the furnace of the instrument followed the defined temperature program (25–1000 ºC), and the Pyris Manager software recorded the sample weight loss and heat flow. The zeta potential of all the three nanocomposites were measured by Zetasizer Nano ZS (Malvern, UK). About 5 mg of sample was mixed with 10 ml of PBS buffer (pH 7.4) and sonicated for 30 min. The sample was then transferred into disposable measuring cell and analysed. The measurement was carried out three times at room temperature. Nanocomposites were mixed with the dispersing medium, i.e.,; milli-Q water and carefully filled in the cell of the instrument to the recommended level. Sample loading was done by inserting the filled cell into the instrument’s cell holder, ensuring correct orientation. Then sufficient time of 1–5 minutes was allowed for the sample temperature to equilibrate with the instrument’s set temperature and the measurement run was started. The instrument applied an electric field, measured electrophoretic mobility using electrophoretic light scattering (ELS), and calculated the zeta potential. The hydroxyl groups, polyphenols and amino compounds of samples were confirmed using FT-IR spectroscopy (Perkin Elmer, USA). The sample loading was done by placing a small amount (a few milligrams) directly onto the crystal of the instrument. Distributed it evenly to cover the crystal’s surface and consistent pressure was applied using the ATR’s clamping mechanism and the sample was run to determine the functional moieties of synthesized nanocomposites.

The morphological features of Ag-*MO* and Ag-Zn-*MO* were measured using SEM-EDS using JSM-6610LV from JEOL Ltd, Tokyo, Japan. Elemental mapping was obtained by energy dispersive spectroscopy (EDS) using Aztec software (oxford). The samples were measured using Gatan digital micrograph software. The material was washed and placed on the stub and after gold coating and it was viewed under SEM [15, 16]. The dynamic light scattering (DLS) analysis of the silver nanoparticles (AgNPs) synthesized using *Moringa oleifera* leaf extract was also performed to check the size of the NPs.

XPS (Omicron ESCA-II SR with Mg-Ka source 1253.6 eV) using Mu metal analysis chamber X-ray radiation Turnkey computer controlled UHV system fast entry load lock with multi sample entry camera assisted sample navigation was also conducted for the identification of the chemical and oxidation states of both the nanocomposites. The silver content was determined using an inductively coupled plasma optical emission spectrometer (ICP-OES) Horiba ULTIMA 2 instrument (31,32).

### 2.4. Antifungal studies of Ag-*MO* and Ag-Zn-*MO*

#### 2.4.1. Growth media and strain.

During this study, *C. auris* CBS10913T strain (Clade II) was used for the antifungal studies [16]. Prior to the experiment, the *C. auris* strain was resurrected in YPD broth that contained 1% (w/v) yeast extract, 2% (w/v) dextrose, and 2% (w/v) peptone, along with 2% (w/v) agar for the plates. Every strain of *Candida* was kept at −80 °C in 30% (v/v) glycerol stocks.

#### 2.4.2. Broth dilution assay.

The Clinical and Laboratory Standards Institute’s (CLSI) method M27-A3 guidelines were adhered [17] for determination of MIC with slight modifications. On a 96 well plate, 100 ul of YPD media was added to each well which was followed by addition of Ag-*MO* and Ag-Zn-*MO* and then diluted serially. 100 ul of *C. auris* cell suspension was added to each well and then finally OD_600_ was measured after 48 hrs of incubation at 30° C.

#### 2.4.3. Spot assay and Static/Cidal assay.

Drop dilution assay was used for accessing spot assay [18]. Yeast extract peptone dextrose (YPD) agar plates containing nanocomposites Ag-*MO* and Ag-Zn-*MO* at their MIC_90_ concentrations, i.e., 125 μg/mL and 250 μg/mL and control plates (without compounds) were prepared. In spot assay, 5 μl of *Candida* cell culture (0.1 OD_600_) was applied to these plates in presence as well as absence of compounds, using fivefold serial dilutions. The plates were incubated at 30°C for 48 hours, and growth differences were recorded.

For static/cidal assay, *C. auris* ((0.1 OD_600_) cells were initially grown overnight at 30°C in the presence of Ag-*MO* and Ag-Zn-*MO*, each at its minimum inhibitory concentration (MIC). The next day, 100 μl of these cultures were transferred to fresh YPD medium (lacking Ag-*MO* and Ag-Zn-*MO*) and incubated again overnight at 30°C. Finally, the following day, the growth of the *C. auris* cultures was assessed by measuring their optical density at 600 nm (OD_600_) using a spectrophotometer [19].

#### 2.4.4. Checkerboard titration method for drug synergism of Ag-*MO* and Ag-Zn-*MO* with known antifungal drugs (FLU, CAS, AMP B).

Checkerboard assays were conducted to evaluate the potential for synergistic, antagonistic, or indifferent interactions between both the nanocomposites and fluconazole (FLU), caspofungin (CAS) and amphotericin B (AMP B). Prior to testing, stocks and serial dilutions were made in accordance with the National Committee for Clinical Laboratory Standards (NCCLS) [17]. Except for the first and last two columns, 100 μL of YPD (Yeast Extract Peptone Dextrose) media was added to each well of a 96-well microdilution plate. Next, 200 μL of the compounds and medium were added to the first column. The final two columns were used as positive controls, which contained *Candida* cells, and negative controls, which contained only medium. While the second antifungal medication was serially diluted horizontally, the first ingredient in combination was diluted serially and vertically. 100 μL of *Candida* cells (0.1 OD_600_) were added to all the wells, and the wells were then incubated for 48 hours at 30°C. Each combination of two medications was organized according to the checkerboard design, with the maximum concentrations of each drug in opposite corners. The lowest antibiotic concentration that, as seen with the naked eye, totally stopped the organism’s development [19].

#### 2.4.5. Effect on Biofilm formation.

*C. auris* biofilms were grown in 96-well tissue culture plates. To start, 100 µl of a 1.0 x 10⁶ cells/ml *C. auris* suspension in RPMI 1640 medium was added to each well. The plates were then left undisturbed at 37°C. After 90 minutes, allowing cells to adhere, the liquid was removed and any unattached cells were washed away. Fresh medium was then added, and the plates were incubated at 37°C for an additional 24–48 hours to allow mature biofilms to form [18].

**Crystal Violet staining of biofilm.**

Briefly, *C.auris* cells were diluted with YPD media and then 200 µl cell suspensions were added to flat-bottomed 96-well microplates. Both the nanocomposites were simultaneously added to the wells of microplates and incubated fat 37^o^C for 24 hrs. After incubation, the planktonic cells were removed from the wells, and the wells were washed with PBS. A 2.3% (w/v) crystal violet solution was then added and allowed to stain the biofilms for 5 minutes at room temperature. The stain was subsequently removed, and the wells were washed twice with PBS. Finally, the stained biofilms were observed under microscope [19].

##### 2.4.5.1. Biofilm metabolic activity

50 μL of 3- [4, 5-dimethylthiazol-2-yl]-2, 5-diphenyltetrazolium bromide (MTT), a tetrazolium salt, was added to wells containing biofilms with and without Ag-*MO* & Ag-Zn-*MO* to measure the metabolic activity of biofilms. MTT, a metabolic indicator, was diluted 1:5 in prewarmed 0.15 M phosphate-buffered saline (PBS) before addition. The wells were incubated at 37°C for five hours. The MTT formazan product, a colored compound formed by metabolically active cells, was solubilized in dimethyl sulfoxide (DMSO). The optical density of the solubilized formazan was then measured at 450 nm to perform quantification. Absorbance values directly correlated with the metabolic activity of the biofilm [18].

##### 2.4.5.2. Quantification of biofilm biomass

To prepare for biofilm growth, sterile silicone squares (1.5 x 1.5 cm) were first pre-weighed and then treated overnight with bovine serum. After washing with PBS, they were placed into individual wells of a 12-well plate. *C. auris* cells, taken from their exponential growth phase, were diluted to an OD₆₀₀ of 0.2 in Spider medium and added to each well containing a silicone square. These were incubated for 90 minutes at 37°C with gentle agitation (150 rpm) to allow initial cell adhesion. Non-adherent cells were then removed by washing the squares with 2 ml of PBS. Subsequently, the squares were transferred to a new 12-well plate, each containing 2 ml of fresh Spider medium along with Ag-*MO* (100 µg/mL) and Ag-Zn-*MO* (200 µg/mL) respectively. Biofilm formation was then allowed to proceed for an additional 60 hours at 37°C with 75 rpm agitation. To measure dry mass, the biofilm-laden silicone squares were washed with PBS, air-dried, and weighed.

Each biofilm’s total biomass was calculated by deducing its pre-weighed weight from the weight of the silicon square following biofilm formation [18].

#### 2.4.6. Cell adherence assay.

Adherence assays were carried out in accordance with prior study [18]. After being grown on YPD media for the entire night at 37^o^ C, the yeast cells were resuspended in 2 milliliters of sterile PBS and centrifuged twice before being resuspended in spider media. Epithelial cells were obtained from the cheek mucous membrane of a co-author voluntarily by gently scraping sterile cotton swabs which were then gently agitated and centrifuged at 3000 rpm for three minutes each to wash with PBS. In order to conduct adherence tests, 1 milliliter of each suspension was combined in a test tube. This was followed by two hours of gentle stirring at 37^o^C in the presence of Ag-*MO* and Ag-Zn-*MO* separately. Following incubation, each test tube received two drops of 0.4% trypan blue solution, and the mixture was gently mixed. Finally, 10 ul stained suspension was examined under a microscope [19].

#### 2.4.7. Cell wall staining and analysis.

Exponential-phase *C. auris* cells (2.5 × 10⁶ cells) were obtained from overnight cultures of control, Ag-*MO*, and Ag-Zn-*MO*- treated samples. The cells were then fixed for 30 minutes using 4% paraformaldehyde in phosphate-buffered saline. Fixed cells were washed and stained with ConA-fluorescein conjugate (50 μg/mL) for mannan, AB (100 μg/mL) for β-1, 3-glucan, and CFW (5 μg/mL) for chitin. For half an hour, the staining was done at room temperature. Finally, the fluorescence-stained cells were seen at 100 X magnification using a Nikon fluorescent microscope. NIS Elements program was used to take pictures [18].

#### 2.4.8. Propidium Iodide Uptake.

Propidium Iodide (PI) is a fluorescent dye that glows when it binds to nucleic acids. It cannot easily pass through the membranes of living cells. PI is used to tell the difference between both healthy and injured *Candida* cells. *Candida* cells (0.1 OD_600_) were inoculated with Ag-*MO* and Ag-Zn-*MO* and were allowed to grow by gentle shaking for 3 hours. After that, the cells were cleaned, centrifuged, and stained for 15 minutes with PI (1 μg/mL). The cells were then examined using a fluorescent microscope [18].

#### 2.4.9. Estimation of Ergosterol.

0.1 OD_600_ *C. auris* cells were inoculated in 50 ml of YPD in the presence of Ag-*MO* and AgZn-*MO*. The cells were treated with a solution of potassium hydroxide (KOH) dissolved in alcohol. The strong alkaline conditions of KOH help to disrupt cell membranes and release the sterols and %age of the wet weight of the cells was calculated to estimate ergosterol content by following equation:

% Ergosterol + % 24(28)-DHE= [A281.5/290). F]/ pellet weight; 24(28)-DHE= [A230/518). F]/ pellet weight and % Ergosterol = [% ergosterol + % 24(28) DHE]—% 24(28) DHE, where F is the factor for dilution in petroleum ether and 290 and 518 are the E values (in percent per centimeter) determined for crystalline ergosterol and 24(28)-DHE, respectively [18,19].

#### 2.4.10. Vacuole acidification & morphology study.

Untreated cells (used as control); Ag*-MO* & Ag-Zn-*MO* were overnight cultured in YPD media. Following a 4-hour suspension in fresh YPD, cells were washed in 1x PBS and FM4–64 (a fluorescent lipophilic, styryl dye) at 40μM was introduced, and further incubation was done at 30°C for 15 minutes. Washing and resuspension in newly prepared YPD was repeated after incubating at 30°C for 60 minutes. Lastly, pictures were taken at a magnification of 100x using a fluorescent microscope [18].

#### 2.4.11. R6G extracellular efflux assay.

A previously established technique was used to determine the efflux of R6G [18]. In short, a culture of yeast that had grown overnight was pelleted, cleaned, and resuspended in PBS without glucose, resulting in about 1 x 10^6^ cells. After being de-energized in 2-DOG and 2, 4 DNP for 45 minutes, the cells were cleaned and reconstituted in PBS devoid of glucose. After adding R6G to a final concentration of 10 μM, it was incubated at 30°C for 40 minutes. To evaluate efflux, the equilibrated cells were cleaned and resuspended in PBS devoid of glucose. One milliliter of samples was taken out at the designated intervals and centrifuged for 2 minutes at 9000 x g. After collecting supernatant, the optical density (OD) at 527 nm was determined. The cells reconstituted in PBS devoid of glucose were supplemented with 2% glucose to determine energy-dependent efflux. Every experiment had glucose-free negative controls and positive control had only untreated *Candida* cells with glucose and R6G [19,20]. The time intervals when readings were taken were 10 minutes apart. For competition assays, Ag-*MO* (100 μg/mL) and Ag-Zn-*MO* (200 μg/mL) were added respectively to the de energized cells 30 min before the addition of R6G at various concentrations (10 μM, 20 μM, 30 μM, 40 μM) and allowed to equilibrate to follow glucose for energy-dependent efflux as above. Various concentrations of R6G were added in order to determine the rate of the reactions to get the slope for competitive or non-competitive plots.

#### 2.4.13. *Caenorhabditis elegans* studies.

*Caenorhabditis elegans* (*C. elegans)* were infected with *C. auris* and monitored for survival. To evaluate the antimycotic effects of both the nanocomposites, *C. elegans* were co-cultured in presence or absence of Ag-*MO* and Ag-Zn-*MO* with *C. auris* at their sub-MIC concentrations which are 100 μg/mL and 200 μg/mL respectively. Intestinal persistence was visualized using fluorescence microscopy. Survival studies of *C. elegans* infected with *C. auris* were conducted using the Kaplan-Meier method. Approximately 50 young adult female *C. elegans* worms in the L4 developmental stage were transferred from a culture of *E. coli* OP50 bacteria to BHI growth medium supplemented with Ag*-MO* (100 μg/mL) and Ag-Zn-*MO* (200 μg/mL). *C. auris* was introduced to worms and their survival was tracked daily for 7 days. Images were obtained on the seventh day of infection, and the infected *C. elegans* were cultured at 25 °C for seven days, scoring as live or dead every day. To visualize *C. auris* in the worm intestine, fluorescence microscopy was used. Images were captured using a stereo microscope and a fluorescence microscope [18].

#### 2.4.14. Macrophage killing & infection.

THP-1 cells, a type of human white blood cell, were grown in a nutrient-rich medium. The cells were treated with 15 nM Phorbol 12-myristate 13-acetate (PMA), a pharmacological stimulant, for 48 hours before being rinsed thrice. Afterward, these macrophage-like cells were infected with *C. auris*. This fungus was prepared by centrifuging a culture, resuspending it in the same growth medium, and breaking up any clumps with a syringe. The infection was carried out at a high fungal-to-cell ratio (5:1) (pathogen: host) for 4 hours. Following the infection, the cells were treated with an antibiotic, amikacin (200 µg/ml). Following a PBS wash, the cells were resuspended in RPMI with 10% FBS added, and the remaining intracellular fungi were allowed to grow for 24 hours. After lysing the cells with 0.5% Triton-X, the cell lysate was serially diluted in triplicate and plated on agar plates to count the number of surviving fungi. The number of fungal colonies that grew on the plates was counted to assess the effectiveness of the treatment [21].

#### 2.4.15. Hemolytic activity assay.

A modified version of a previously established approach was used to determine the hemolytic activity of Ag-*MO* and Ag-Zn-*MO* [18]. The absorbance of hemoglobin at 540 nm was used to quantify its discharge into the supernatant. PBS was used as a negative control, while 1% Triton X-100 was utilized as a positive control. The percentage of hemolysis was calculated based on the absorbance of the sample, the blank, and the Triton X-100-treated sample.


Hemolysis (%) = [(Asample − Ablank)/ (ATriton –A blank)] *100

#### 2.4.16. Statistical analysis.

All experiments were conducted in triplicate (n = 3). Student’s t-test was used to examine the data, and P < 0.05 was deemed statistically significant. The mean ± standard deviation is used to express the data.

## 3. Results

### 3.1. Characterization of biosynthesized silver nanocomposites on *MO*

The first sign of silver nanoparticle formation was the shift in color from pale yellow to a deep brown. This color change indicates the reduction of silver ions to silver nanoparticles, and it also confirms that *MO* extract solution played an important role in conversion of silver ions into silver nanocomposites due to the reducing compounds present in it. The physicochemical characteristics of nanocomposites affect their efficacy, biodistribution and behavior. Therefore, evaluating the functional characteristics of nanomaterials depends on the characterization of metallic nanoparticles. Techniques for characterizing NPs include zeta potential, scanning electron microscopy (SEM), X-ray diffractometry (XRD), Fourier transform infrared spectroscopy (FTIR), and UV-vis spectroscopy etc. Combining these spectroscopic methods allows for the determination of the quality of the synthesized nanomaterials and provides information that would be impossible to obtain using only one method.

#### 3.1.1. X-ray diffraction.

**Fig 1:**
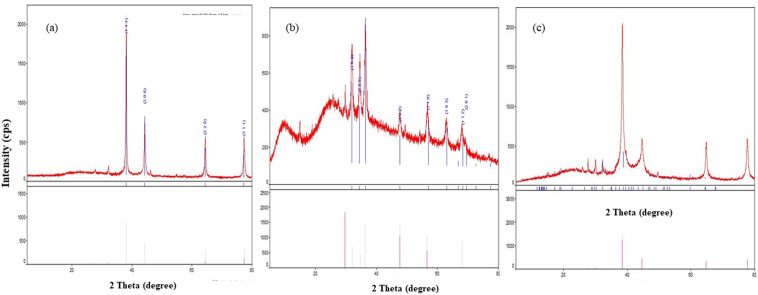
XRD pattern of Ag-*MO* and Ag-Zn-*MO.* a) Peaks (111), (200), (220) and (311) planes correspond to face centered cubic lattice of AgNPs. b) XRD pattern of ZnO NPs. (100), (002), (101), (102), (110), (103), (200), (112) and (201) planes show hexagonal wurtzite structure in pure phase. c) XRD pattern of Ag-Zn-*MO*. Pattern exhibits peaks corresponding to both face-centered cubic structure of AgNPs and hexagonal shaped wurzite ZnONPs. Such crystallinity of AgNPs indicates the deposition of metallic phase of Ag on the surface of ZnO nanoparticles.

X-ray Diffraction (XRD) is very useful in determining the crystal structure, atomic arrangement and crystal size. Fig 1a shows the X-ray diffraction pattern of AgNPs derived from *MO* leaves. The nanoparticle showed a sharp high intensity peak indexed to (111), (200), (220) and (311) planes corresponding to face centered cubic lattice of AgNPs. The absence of broad peaks shows the existence of pure form of AgNPs without *Moringa* plant extract residues. Fig 1 b shows the X-ray diffraction pattern of ZnO NPs derived from *MO* leaves. The formation of hexagonal wurtzite structure in pure phase has been confirmed with the distinct planes corresponding to (100), (002), (101), (102), (110), (103), (200), (112) and (201), respectively [22]. The presence of two additional amorphous peaks at lower 2 theta range below 30° could be attributed to the residues of *MO*. The peaks confirm the deposition of plant extracts on the surface of nanoparticles and such composites clearly show the role of antioxidants, flavonoids and associated organic contents in the formation of ZnO nanoparticles [22]. Fig 1 c shows the X-ray diffraction pattern of Ag-ZnO NPs derived from *MO* leaves. The XRD pattern exhibits peaks corresponding to both face-centered cubic structure of AgNPs and hexagonal shaped wurzite ZnO. AgNPs peaks corresponding to (111), (200), (220) and (311) planes were dominant, while less intense peaks of wurtzite structure of ZnO were visible at lower 2 theta values. Such large crystallinity of AgNPs indicates the deposition of metallic phase of Ag on the surface of ZnO nanoparticles.

#### 3.1.2. Zeta potential measurement.

**Fig 2:**
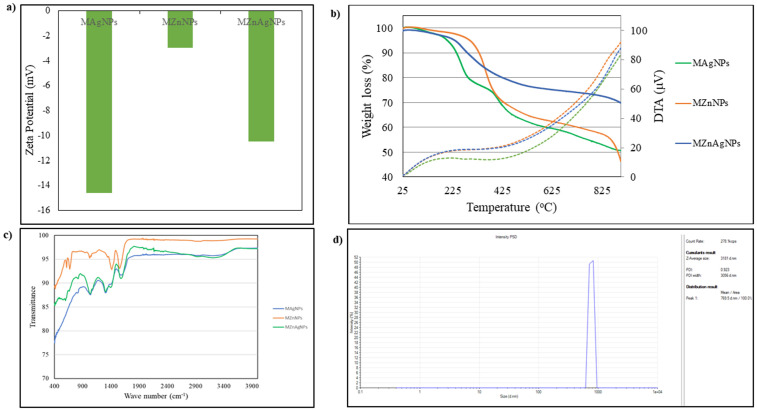
Zetapotential of Ag-*MO* and Ag-Zn-*MO.* **a) Ag-*MO* showed the highest negative zeta potential of −14.6 mV, while ZnO nanoparticles showed the lower negative zeta potential of −3.0 mV while Ag-Zn-*MO* showed the intermediate zeta potential of −10.5 mV. b) thermogravimetric analysis of Ag, ZnO and Ag-ZnO NPs derived from *Moringa oleifera* leaves.** Ag-MO showed three distinct weight losses between 100−280 °C, 300−500 °C and 500−900 °C with total weight loss of about 50%. In Ag-Zn-MO, the weight loss was reduced to 30% with a distinct decomposition trend. ZnO alone showed a broad decomposition weight loss starting from 100−440 °C and second weight loss from 450−900 °C with total weight loss of about 53%. **c)** FTIR spectra of Ag-*MO*, ZnO and Ag-Zn-*MO*. The spectra of Ag-*MO* and Ag-Zn-*MO* showed a distinct broad peak at about 3245 cm^-1^ corresponding to the hydroxyl groups polyphenols and amino compounds. The peak at 1606 cm-1 is ascribed due to the carbonyl stretching functional group of amides. **d)** DLS data and size distribution graph of AgNPs derived from *Moringa oleifera* leaf extract.

Zeta potential measurement shows the surface charge measurements. Fig 2 shows the zetapotential of Ag, ZnO and Ag-ZnO NPs derived from *Moringa Oleifera* leaves. The technique is useful to study the zeta size and colloidal state stability of synthesized Ag, ZnO and Ag-ZnO NPs. AgNPs showed the highest negative zeta potential of −14.6 mV, while ZnO nanoparticles showed the lower negative zeta potential of −3.0 mV. The composite of Ag-ZnO showed an intermediate negative zeta potential of −10.5 mV. The present study shows that AgNPs and Ag-ZnO NPs possess dispersion stability than ZnO nanoparticles. This is in accordance with a prior study where the presence of capping agent on NPs surface was responsible for repulsive force between NPs that hindered their aggregation [23,24].

#### 3.1.3. *Thermogravimetric analysis.*

Thermogravimetric Analysis (TGA) is used in determining physical phenomena like thermal decomposition and phase transition based on measuring the mass of substance after heat treatment on elevated temperatures. Fig. 2 b shows the thermogravimetric analysis of Ag, ZnO and Ag-ZnO NPs derived from *MO* leaves. AgNPs showed three distinct weight loss between 100–280 °C, 300–500 °C and 500–900 °C with total weight loss of about 50%. ZnO NPs showed a broad decomposition weight loss starting from 100–440 °C and second weight loss from 450–900 °C with total weight loss of about 53%. In the case of Ag-ZnO NPs, the weight loss was reduced to 30% with a distinct decomposition trend observed. For instance, a loss of less weight was observed between 100–280 °C followed by a gradual weight loss up to 900 °C. For the three samples, the observed weight at about 100 °C was ascribed due to moisture adsorbed on the surface of Ag, ZnO and Ag-ZnO nanoparticles. The significant weight loss observed up to 700 °C is ascribed due to the disintegration of phytocomponents of plant extracts [25], in our case it is related to the *MO*.

#### 3.1.4. FTIR analysis.

FTIR is used to determine the surface chemistry of synthesized nanocomposite. The functional groups of biosynthesized Ag-*MO* and Ag-Zn-*MO* nanocomposites were determined by FTIR analysis. Fig 2 c shows the FTIR spectra of Ag, ZnO and Ag-ZnO NPs derived from *MO* leaves. The spectra of Ag and Ag- ZnO showed a distinct broad peak at about 3245 cm^-1^ corresponding to the hydroxyl groups polyphenols and amino compounds [25]. The peak at 1606 cm-1 is ascribed due to the carbonyl stretching functional group of amides. In the case of ZnO, a characteristic stretching peak of Zn-O was observed at about 670 cm^-1^ [26]. The presence of such band at about 665 cm^-1^ in Ag-ZnO shows the composite formation between Ag and ZnO NPs. [27,25] (Fig 2 c).

Dynamic light scattering analysis (DLS) was also done to check the size of the nanoparticles. The results indicate that the average hydrodynamic particle size is approximately 769.5 nm, suggesting possible nanoparticle aggregation in the aqueous medium (Fig 2d). This observation is consistent with the biological synthesis route, which often leads to surface-bound phytochemicals contributing to increased particle size and agglomeration.

#### 3.1.5. SEM analysis.

**Fig 3:**
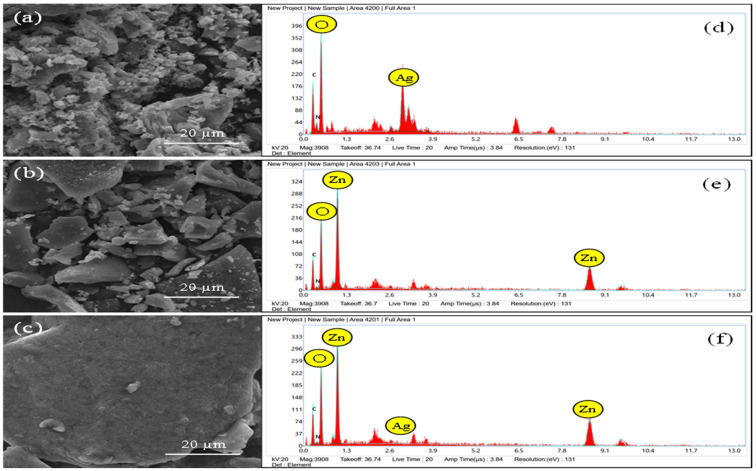
SEM images and EDX spectra. a)** Mixed large, aggregated nanoparticles covered with small, layered particles of AgNO3-***MO*. b)** Formation of variable chucks in ZnO-MO nanocomposite. **c)** Aggregation of small size Ag nanoparticles on microforest flower ZnO structure. **d)** Distinct peak shows AgNO3 reduced to Ag NPs. **e)** Peak shows reduction of ZnO to Zn NPs. **f)** Cohabitation of Ag and Zn after reduction.**

SEM analysis was also performed to determine the morphology of Ag-*MO* and Ag-Zn-*MO* nanocomposite. Fig 3 (a-c) shows the SEM image of Ag, ZnO and Ag-ZnO NPs using *MO* leaf extract. In our study, the chemical precursor was found to play a key role in the formation of different shaped Ag, ZnO and Ag-ZnO NPs. For instance, metal precursor sources such as silver nitrate, zinc acetate and mixed metal precursors tend to form variable morphological features of nanoparticles (Fig 3 (a-c) Ag NPs using silver nitrate precursor indicating formation of mixed large, aggregated nanoparticles covered with small, layered particles (Fig 3a). In the case of green ZnO NPs, the formation of chucks occurs with variable sizes (Fig 3b). Bimetallic Ag and low density ZnO nanocomposite formation tends to form aggregation of small size Ag nanoparticles on microforest flower ZnO structure [27]. In this case, the mixed precursor showed better formation of large morphology of Ag-ZnO indicating an effective bioreduction by *MO* (Fig 3 c). Fig 3(d-f) shows the EDX spectrum of Ag, Zn and Ag-Zn NPs. The formation of distinct Ag and Zn peaks indicates the successful reduction of AgNO_3_ to Ag NPs and Zn acetate to Zn NPs, respectively (Fig 3d and e). Fig 3f shows the synergistic presence of both Ag and Zn NPs indicating cohabitation of both types of nanoparticles formation in a single pot facilitated by *MO* leaf plant reduction [27,28].

Additionally, XPS was also carried out to observe the presence of different metallic peaks that has been presented as a supplementary file. The study of elemental composition and oxidation states of Ag-*MO* and Ag-Zn-*MO* were analyzed which revealed the presence of carbon and oxygen that might have come from residual of *Moringa* extract used in the preparation of nanocomposites. These results confirm the formation of metallic AgNO_3_ without any presence of AgO in Ag-*MO* and the formation of ZnOH and other zinc oxide derivates in Ag-Zn-*MO* [29–32] (Supplementary file, S1 and S2 File).

### 3.2. Ag-*MO* and Ag-Zn-*MO* display considerable antifungal action on *C. auris* with negligible hemolytic activity

**Fig 4:**
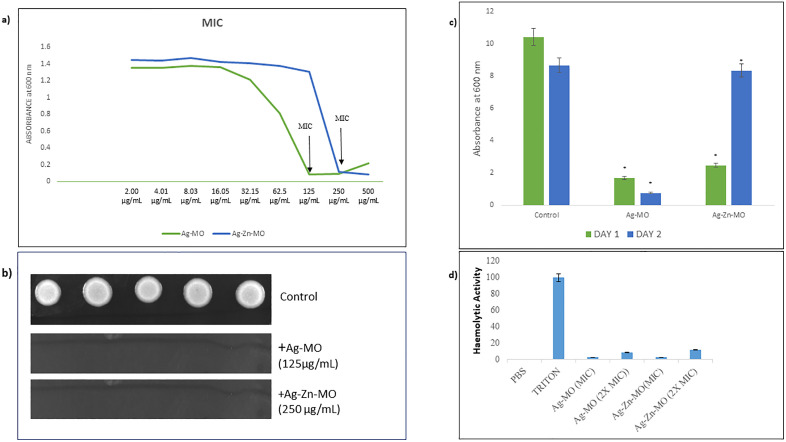
Antifungal activity of Ag-*MO* and Ag-Zn-*MO* (a) Broth microdilution assay with *Candida auris* (CBS10913T) b) Spot assay with *C. auris* in presence of Ag-*MO* and Ag-Zn-*MO* and absence (control) of both. c) Static cidal assay of Ag-*MO* and Ag-Zn-*MO*. Bar graph depicting the revival of untreated candidal cells (Control) and non-revival of Ag-*MO* and Ag-Zn-*MO* treated (MIC _90_) cells in normal YPD media confirming the fungicidal nature of Ag-MO and fungistatic nature of Ag-Zn-MO. d) Hemolytic activity of Ag-MO (MIC, 2X MIC) and Ag-Zn-MO (MIC, 2X MIC) as percentage on Y-axis in comparison to positive control Triton X 100.

To investigate the antifungal ability of both nanocomposites, we conducted two different assays: microbroth dilution assay and spot assay. We observed that the MIC_90_ of Ag-*MO* and Ag-Zn-*MO* against *C. auris* was 125 μg/mL and 250 μg/mL respectively (Fig 4 a). To confirm the results of broth microdilution assay, we performed a spot assay. As expected, no fungal growth was observed at the MIC concentrations of Ag-*MO* and Ag-Zn-*MO* (Fig 4 b) (Supplementary file, S6 File). To determine whether Ag-*MO* and Ag-Zn-*MO* were fungicidal or fungistatic in nature, we conducted a spectrophotometric assay. At the MIC_90_ concentration of Ag-*MO*, fungal growth was inhibited on day 1. However, when the culture was reinoculated on day 2 in absence of Ag-*MO*, no growth was observed, indicating a fungicidal effect (Fig 4 c). In contrast, Ag-Zn-*MO* exhibited a fungistatic effect, as fungal growth resumed on day 2 (Fig 4 c) (Supplementary file, S9 File). We also assessed the effect on *C. auris* cell viability at their respective minimum inhibitory concentrations (MICs). Both nanocomposites significantly reduced cell viability, with Ag-*MO* and Ag-Zn-*MO* causing a 17% and 25% decrease, respectively, compared to the control (Supplementary file, S4 File). We further assessed the potential of both Ag-*MO* and Ag-Zn-*MO*, to damage red blood cells (erythrocytes). This hemolytic assay demonstrated that both nanocomposites exhibited negligible hemolytic activity up to two times their minimum inhibitory concentration (2x MIC). At this concentration, Ag-*MO* induced only 9% hemolysis, while Ag-Zn-*MO* caused 12% hemolysis. In contrast, the positive control, Triton X, led to 100% hemolysis (Fig 4d) (Supplementary file, S8 File). Additionally, *C. auris* cells growth in presence of Ag-*MO* and Ag-Zn-*MO* using a 600 nm optical density (OD) measurement was also studied to predict the subinhibitory concentrations to conduct further biochemical assays (Supplementary file, File S 3).

### 3.3. Ag-*MO* and Ag-Zn-*MO* display drug synergism with known antifungal drugs

The checkerboard microdilution assay revealed that Ag-*MO* and Ag-Zn-*MO* exhibited synergistic interactions with major antifungal drugs, caspofungin, amphotericin B & fluconazole. This was indicated by a fractional inhibitory concentration index (FICI) of < 0.5 (Table 1).

**Table 1:** FICI by checkerboard assay. Drug synergism activity of Ag-*MO* and Ag-Zn-*MO* to the FLU (azole), CAS (echinocandin) and AMP B (polyene).

	FLU	AMP B	CAS
FIC (Ag-MO)	0.38	0.5	0.1
FIC (Ag-Zn-MO)	0.157	0.1	0.31

### 3.4. Ag-*MO* and Ag-Zn-*MO* alter the cell wall polysaccharide composition

**Fig 5:**
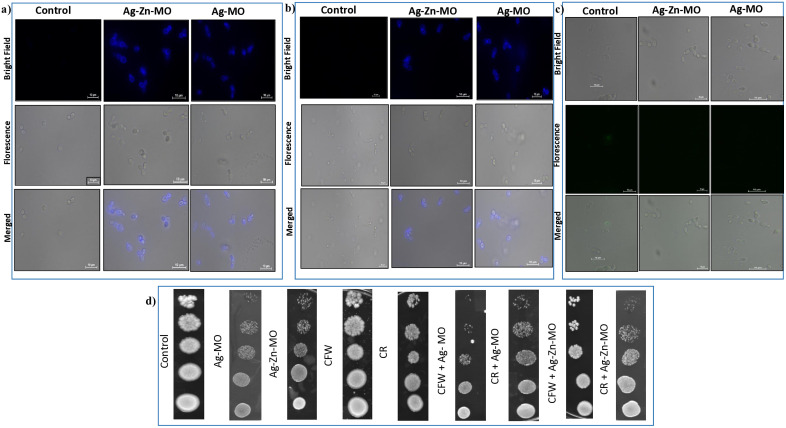
Effect of Ag-*MO* and Ag-Zn-*MO* on cell wall. (a) Chitin (CFW staining) (b) β1,3-glucan (AB staining) (c) mannan (ConA staining) d) Spot assay of control, Ag-*MO* and Ag-Zn-*MO* in presence of CR (10 μg/mL) and CFW (10 μg/mL) for sensitivity to cell wall perturbing agents.

To further investigate the mechanism of action of Ag-*MO* and Ag-Zn-*MO*, we examined their impact on the cell wall components of *C. auris*. Chitin, β-1,3-glucan, and mannan, key components of the fungal cell wall, were stained with specific fluorescent dyes (CFW, AB, and ConA-fluorescein conjugate, respectively). Compared to control cells, cells treated with Ag-*MO* and Ag-Zn-*MO* exhibited increased fluorescence intensity for chitin and β-1,3-glucan stains, suggesting an alteration in their cell wall composition (Fig 5 a, b). Conversely, mannan staining was significantly reduced in treated cells, indicating a potential disruption in mannan synthesis or localization (Fig 5 c). These findings were further corroborated by a phenotypic susceptibility spot assay, which revealed sensitivity of *C. auris* to both Ag-*MO* and Ag-Zn-*MO*, as well as to cell wall stressors like CFW and CR (Fig 5 d).

**Fig 6:**
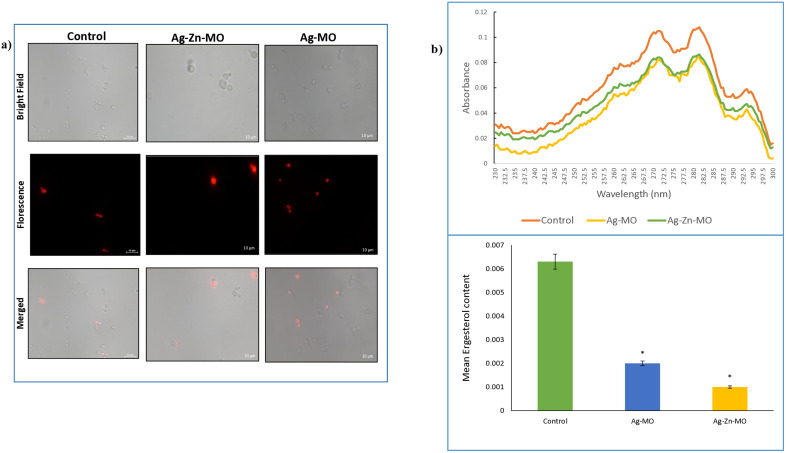
Effect of Ag-*MO* and Ag-Zn-*MO* on membrane integrity. a) Propidium iodide (PI) staining of *C. auris* cells in the presence and absence of Ag-*MO* and Ag-Zn-*MO*. b) Upper panel showing UV spectrophotometric ergosterol profiles of *C. auris* scanned between 230 and 300 nm in the absence and presence of Ag-*MO* and Ag-Zn-*MO*. Lower panel showing relative percentages of ergosterol content absence and presence of Ag-*MO* and Ag-Zn-*MO*. Mean of % ergosterol levels is calculated and normalized by considering the untreated control as 100 ± SD of three independent sets of experiments are depicted on y-axis and * depicts *P < *0.05.

Subsequently, we assessed membrane integrity by measuring propidium iodide (PI) uptake. Increased PI fluorescence indicated compromised membrane integrity (Fig 6 a). To further investigate the impact of Ag-*MO* and Ag-Zn-*MO* on fungal cell integrity, we measured the sedimentation rate of *C. auris* cells using a spectrophotometric test in the presence of Ag-*MO* and Ag-Zn-*MO*. We observed an increased sedimentation rate in the presence of Ag-*MO*, indicating a potential disruption of cell wall integrity and increased cell fragility. Ag-Zn-*MO* also exhibited a moderate effect on sedimentation rate, suggesting considerable but lesser impact on cell wall integrity as compared to Ag-*MO* (Supplementary file, S5 File) (18).

### 3.5. Ag-*MO* and Ag-Zn-*MO* confers ergosterol reduction and inhibits the ABC family efflux activity

**Fig 7:**
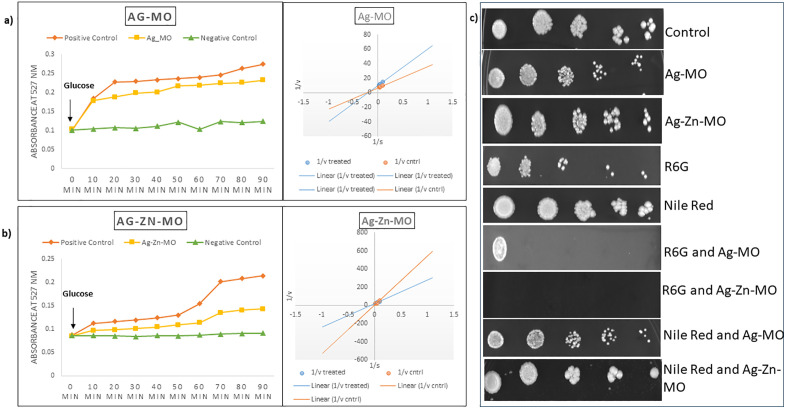
Effect of Ag-*MO* and Ag-Zn-*MO* on efflux pump activity. a) and b) Efflux pump activity of Ag-*MO* and Ag-Zn-*MO*. Left panel shows Extracellular R6G concentrations in control (without Ag-*MO* and without Ag-Zn-*MO*), Ag-*MO* and Ag-Zn-*MO* treated *C. auris* cells. Right panel shows Lineweaver-Burk plot in the presence of Ag-*MO* and Ag-Zn-*MO*. The x-axis (1/S) represents the various concentrations (μM) of R6G used, and the y-axis (1/V) shows the rate of release of R6G in the presence of Ag-*MO* and Ag-Zn-*MO*. c) Phenotypic susceptibility assay of R6G and Nile Red in the presence and absence of Ag-*MO* and Ag-Zn-*MO.*

To further study the effect of Ag-*MO* and Ag-Zn-*MO* on cell membrane, we found that both the nanocomposites disrupt cell membrane integrity by checking their ergosterol levels. We observed a 35% and 50% reduction in ergosterol levels in the presence of Ag-*MO* and Ag-Zn-*MO,* respectively (Fig 6 b) (Supplementary file, S10, S11 Files). Additionally, we found that these nanocomposites inhibited the efflux pump functioning, as evidenced by reduced R6G efflux (Fig 7 a, b). Additionally, we performed a phenotypic susceptibility assay with Nile Red and R6G present. In contrast to Nile Red, we found that growth was suppressed in the presence of Ag-*MO* and Ag-Zn-*MO*, and R6G (Fig 7 c). We were further motivated to investigate the type of inhibition by both nanocomposites by the suppressed R6G efflux. So, mechanistic insights into the mode of inhibition via Lineweaver-Burk plot was done. As the apparent Km increased while Vmax stayed constant, these kinetic investigations demonstrated that both Ag-*MO* and Ag-Zn-*MO* competitively limit R6G efflux by ABC transporters (Fig 7 a, b) (Supplementary file, S12, S13, S14, S15 File).

### 3.6. Vacuolar homeostasis is disrupted in presence of Ag-*MO* and Ag-Zn-*MO*

**Fig 8:**
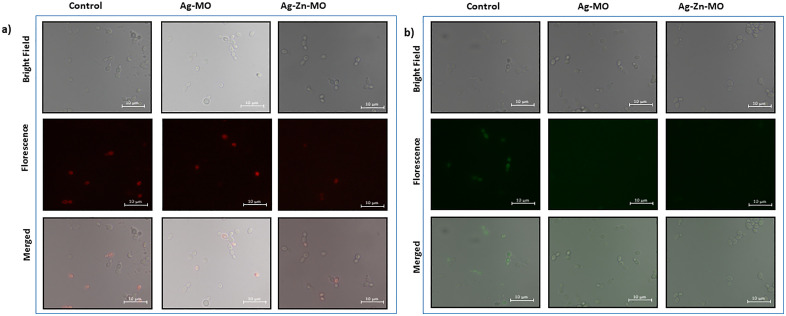
Effect of Ag-*MO* and Ag-Zn-*MO* on Vacuolar homeostasis. (a) Vacuolar morphology of control, Ag-*MO* and Ag-Zn-*MO* treated *C*. *auris* observed by FM4-64 staining. Scale bar = 10 μm. (b) Vacuolar acidification of control, Ag-*MO* and Ag-Zn-*MO C*. *auris* observed using quinacrine staining. Scale bar = 10 μm.

To investigate the impact of Ag-*MO* and Ag-Zn-*MO* on vacuolar function, we assessed both acidification and morphology. FM4–64, a lipophilic dye that is built up in the vacuolar membrane, was used to evaluate vacuolar morphology. Control cells exhibited a regular ring-like staining pattern, indicating normal vacuole morphology. In contrast, cells treated with Ag-*MO* and Ag-Zn-*MO* displayed a diffuse staining pattern, suggesting deformed vacuoles (Fig 8 a). To evaluate vacuolar acidification, we employed quinacrine, a weak base that accumulates in acidic compartments. Control cells exhibited strong quinacrine fluorescence, confirming the acidic pH of their vacuoles. However, Ag-*MO* and Ag-Zn-*MO* -treated cells showed reduced fluorescence, indicating a compromised acidic environment within the vacuole. These findings suggest that Ag-*MO* and Ag-Zn-*MO* disrupt vacuolar homeostasis, potentially affecting various cellular processes (Fig 8 b).

### 3.7. Ag-*MO* and Ag-Zn-*MO* inhibit formation of biofilms and decrease adherence to buccal cells

**Fig 9:**
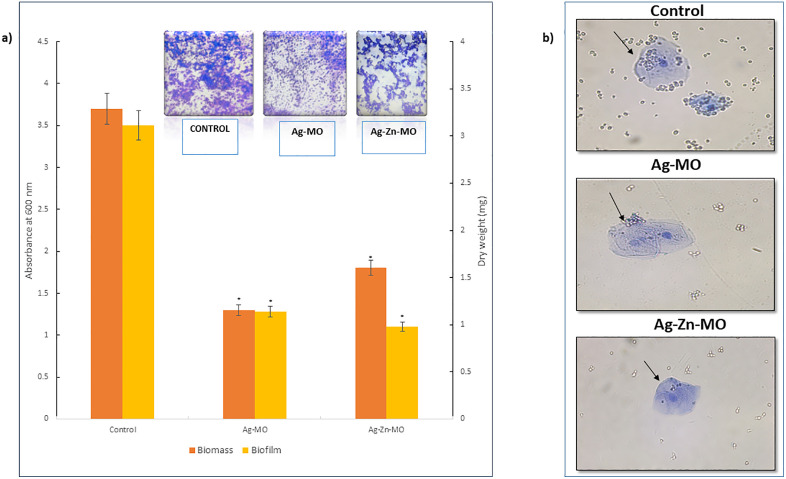
Effect of Ag-*MO* and Ag-Zn-*MO* on biofilm formation. a) MTT assay depicting the disrupted biofilm formation in Ag-*MO* and Ag-Zn-*MO* treated cells by left y axis * depicts *P < *0.05. Depleted biomass measurement of Ag-*MO* and Ag-Zn-*MO* treated cells by right y axis * depicts *P < *0.05. Inset in the graph shows crystal violet stained *C. auris* in Ag-*MO* and Ag-Zn-*MO* treated cells. b) Effect of Ag-*MO* and Ag-Zn-*MO* on cell adherence using buccal epithelial cells showing Inhibition of buccal epithelial cell adhesion by Ag-*MO* and Ag-Zn-*MO* treated cells. Arrows indicating the non-attachment of *C. auris cells* with buccal epithelial cells.

Furthermore, we investigated the effect of Ag-*MO* and Ag-Zn-*MO* on *C. auris* biofilm formation. Qualitative crystal violet (CV) staining revealed impaired development of biofilms in the presence of both nanocomposites. To quantify the impact on biofilm metabolic activity, we employed the MTT assay. This assay showed a significant 84% and 82% reduction in metabolic activity in Ag-*MO* and Ag-Zn-*MO* treated biofilms. Finally, we measured biofilm biomass and observed 70% and 85% reduction in the presence of Ag-*MO* and Ag-Zn-*MO*, confirming their potent biofilm inhibitory properties (Fig 9 a) (Supplementary file, S7 File). We also assessed the impact of Ag-*MO* and Ag-Zn-*MO* on adherence of *C. auris* colonization on human epithelial buccal cells and deciphered that when compared to untreated cells, both nanocomposites disturb the adhesion of fungal cells to buccal cells (Fig 9 b).

### 3.8. Ag-*MO* and Ag-Zn-*MO* enhanced *C. elegans* survival infected with *C. auris*

**Fig 10:**
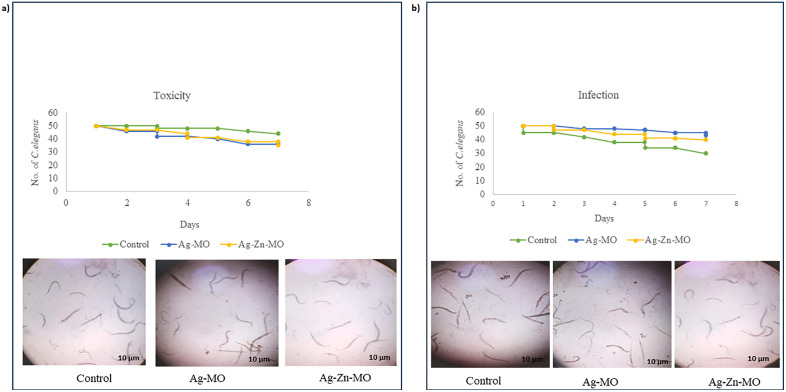
Effect of Ag-*MO* and Ag-Zn-*MO* on nematode survival. a) Toxicity test of Ag-*MO* and Ag-Zn-*MO* on *C. elegans* depicted by the Kaplan–Meier curve showing number of *C. elegans* in the presence of Ag-*MO* and Ag-Zn-*MO* at sub-MIC concentration. Worm survival was determined based on movement. The toxicity of Ag-*MO* and Ag-Zn-*MO* was studied on nematodes by determining survival rates until 7 days. b) Survival of *C. auris*–infected *C. elegans*. Kaplan–Meier curve showing % survival of *C. auris*–infected *C. elegans* in the presence of Ag-*MO* and Ag-Zn-*MO* until 7 days.

**Fig 11:**
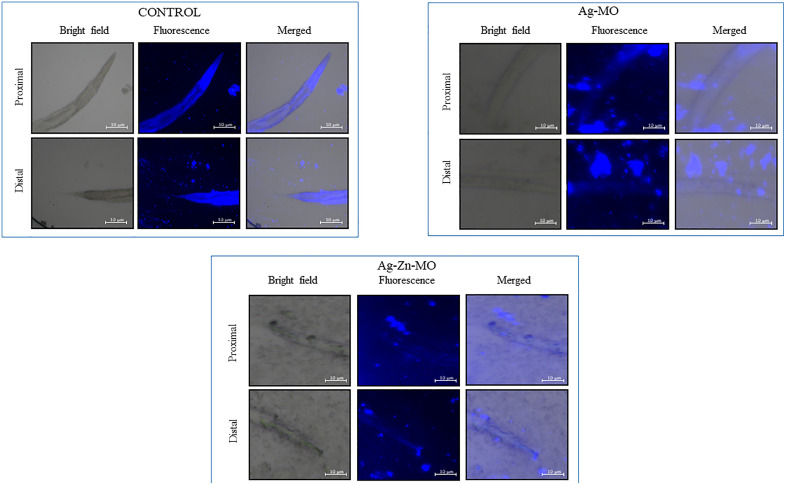
Intestinal persistence of *C. auris* represented by the CFW-stained *C. auris* visualized in intestine of *C. elegans* treated with Ag-*MO* and Ag-Zn-*MO* showing lesser fungal burden. Magnification 100x.

To validate our *in-vitro* findings, we utilized the widely used nematode *C. elegans* to evaluate the *in-vivo* antifungal efficacy of Ag-*MO* and Ag-Zn-*MO*. To determine the potential toxicity of Ag-*MO* and Ag-Zn-*MO* to the host organism, *C. elegans* were exposed to sub-inhibitory concentrations of 100 μg/mL and 200 μg/mL, respectively. Over a 7-day period, there were no discernible variations in worm growth (Fig 10 a). Thus, this nontoxic (sub-MIC) concentration was used for further evaluation. We also evaluated the antimycotic effects of both the nanocomposites. Remarkably, the rate of survival of *C. elegans* increased significantly in presence of both Ag-*MO* and Ag-Zn-*MO* (Fig 10 b). Additionally, *C. elegans* infected with *C. auris* were stained with CFW to visualize fungal persistence. While untreated worms exhibited clear fungal colonization in the intestine, Ag-*MO* and Ag-Zn-*MO* treated nematodes showed significantly reduced fungal burden, as indicated by decreased fluorescence intensity (Fig 11) (Supplementary file, S16 and S17 File).

### 3.9. Ag-*MO* and Ag-Zn-*MO* potentiates macrophage-mediated candidacidal activity

**Fig 12:**
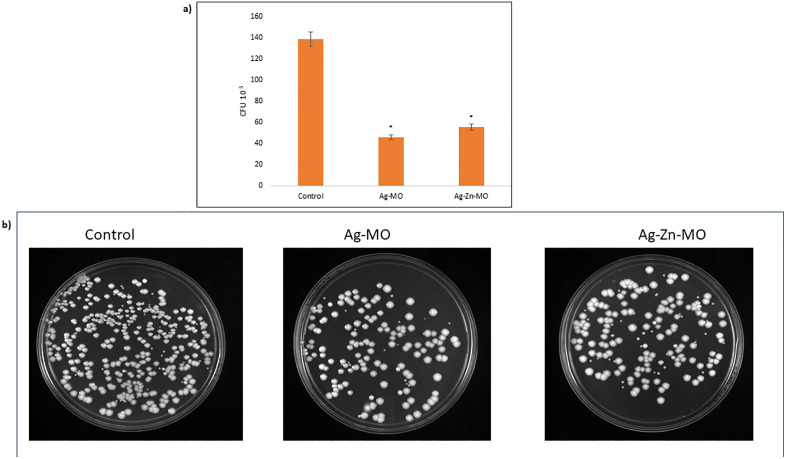
Effect of Ag-*MO* and Ag-Zn-*MO* on macrophage killing ability. a) Bar graph represents CFU log10^5^/ ml on the y-axis after 24-h infection with control (-Ag-*MO* and Ag-Zn-*MO*) and treated (+Ag-*MO* & + Ag-Zn-*MO) C. auris* depicting the killing of fungi by macrophages. Data are expressed as mean ± SD of three independent sets of experiments. b) CFU image of control and treated (+Ag-*MO* & + Ag-Zn-*MO*) *C. auris* after 24-h infection. Magnification 40×.

To further substantiate *in vivo* efficiency, *C. auris* was used to infect macrophages obtained from THP-1. Colony-forming units (CFUs) were used as an indirect indicator of the degree of fungal development within macrophages to evaluate the efficacy of phagocytosis-mediated killing. At the 24-hour time point, *C. auris* treated with Ag-*MO* and Ag-Zn-*MO* demonstrated a significant reduction in intracellular burden, with approximately 46 CFU/ml and 55 CFU/ml compared to 138 CFU/ml in control cells. These findings were further supported by CFU images (Fig 12 a, b) (Supplementary file, S18 File).

## 4. Discussion

Plants, often referred to as nature’s chemical factories, offer a sustainable and cost-effective approach to nanoparticle synthesis. Their ability to produce a vast array of phytochemicals makes them ideal candidates for the eco-friendly production of nanoparticles. Various plant components, including fruits, leaves, stems, and roots, have been widely utilized for the green synthesis of nanoparticles such as silver, zinc oxide, and others. Plant extracts are rich in phytocompounds like polyols, terpenoids, and polyphenols, which play a vital role in the bioreduction of metal ions. Green synthesis methods, employing organisms such as plants, fungi, bacteria, actinomycetes, and yeasts, offer a more sustainable substitute for conventional physical and chemical techniques. This is demonstrated by several recent studies dealing with the green synthesis approach to form various nanomaterials (46). Ali et al. utilized tomato peel extract as a reducing and capping agent to biosynthesize silver nanoparticles (TPE-AgNPs) and evaluated their antimicrobial activity against pathogenic bacteria and fungi [33]. Singh et al. leveraged a leaf extract from *Carissa carandas* L. to achieve the green synthesis of silver nanoparticles [34]. In one such approach, *Moringa Oleifera* leaf extracts were used to reduce, cap, and stabilize silver and zinc ions, leading to the formation of silver nanocomposites and silver-zinc nanocomposites. Notably, *MO* has shown promise in combating fungal infections. It produces the antifungal protein Mo-CBP3, and its hexadecenoic and oleic acids have also been reported to possess antifungal properties [35]. According to a study by Gufran et al, Fourier transform infrared analysis showed the possible interactions between silver and bioactive molecules in *Moringa oleifera* leaves extracts and the phenols, carboxylic acids, proteins, and terpenoids present in *Moringa* leaf extract were likely the agents responsible for both the synthesis and reduction of silver nanoparticles. This study showed potent antifungal as well as antibacterial activity [14]. In addition, the antimicrobial potential of *Moringa*-extract-derived FeONPs has also been demonstrated. Jegadeesan et al. reported that biogenic FeONPs produced from *Moringa* leaves also showed activity against *S. aureus* and *B. subtilis* [36]. Significantly, the manufactured Fe or FeONPs with *MO* leaves exhibited robust antibacterial activity against bacterial strains at lower concentrations than traditional antibacterial medications [36].

Firstly, the two nanocomposites (Ag-*MO* and Ag-Zn-*MO*) capped with *MO* were characterized and the results showed good correlation with other studies. This is in accordance with a study by Vibhuti et al and Gufran Mahmood et al [14,37]. One of the other reasons for color change may be because of presence of surface-active molecules like terpenoids and flavonoids in *MO* leaf extract that makes the nanocomposite more stable. The observed diffraction pattern coincides with the results of Giri et al. (2022). Alharthi et al. (2020) has observed similar trends of XRD in their study and attributed such phenomenon due to the larger ionic radius of Ag^+^ than ZnO species leading to the formation of Ag on the surface of ZnO [27].

Pirtarighat et al. (2018) has reported that negatively charged carboxylate, polar hydroxyl and carbonyl groups interact with the positively charged Ag^+^ leading to the reduction and stabilization of AgNPs. *Moringa* leaves containing various bioactive components including alkaloids, flavonoids, vitamins, minerals etc., have played an important interactive role in reduction of Ag ions [38,39]. Also, Jevapatarakul et al. (2020) has reported that plant extract (*C. formosum*) was not involved in shaping the nanoparticles and solid transformation from zinc hydroxide to zinc oxide nanoparticles. But in our study, the chemical precursor was found to play a key role in the formation of different shaped Ag, ZnO and Ag-ZnO NPs.

To establish novel antifungal agents, we investigated the antifungal potential of two nanocomposites (Ag-*MO* and Ag-Zn-*MO*) capped with *MO* against *Candida auris* and explored their potential mechanism of action. Through standard drug susceptibility testing, we demonstrated the significant anticandidal activity of both the nanocomposites against both *C. auris.* According to one study by Mondal et al, antifungal activity of Ag and Au nanoparticles capped with *MO* against *Aspergillus* sp. was observed at a concentration of 200 mg/ml [40]. But our findings concluded that Ag-*MO* and Ag-Zn-*MO* capped with *MO* exhibited anti-*candida* activity against *C. auris* at 125 μg/mL and 250 μg/mL at their MIC’s respectively. It is well-established that many commonly used antifungal drugs, such as azoles, polyenes, and allylamines, target fungal cell membranes [41]. Interestingly, we explored that only Ag-*MO* was fungicidal at 125 μg/mL as compared to Ag-Zn-*MO* which was found out to be fungistatic at their respective MIC’s. This difference may be because of the difference in composition in Ag-Zn*-MO* where zinc is combined with silver nanocomposite. To combat drug-resistant fungal infections, combination therapy is often employed to enhance the efficacy of existing antifungal drugs. In this research, we investigated the potential synergistic effects of Ag-*MO* and Ag-Zn-*MO* with three major classes of antifungals: fluconazole, amphotericin B, and caspofungin. Our findings indicate that both exhibit synergistic activity with all three classes of antifungals. Also, up to two MICs, both the nanocomposites have very little hemolytic activity against human erythrocyte cells. While “potential toxicity” often carries a negative connotation, the fact that nanocomposites exhibit negligible hemolytic activity is actually a highly desirable and beneficial characteristic in regard to human beings, particularly for biomedical applications like enhanced blood compatibility, reduced risk of thrombosis, reduced systemic toxicity by reducing the initial inflammatory burden on the body, contributing to a more benign interaction with the immune system and improved pharmacokinetics and pharmacodynamics We observed a significant reduction in cell viability of *C. auris* when challenged with the Ag-*MO* and Ag-Zn-*MO* both. These results suggest that both the nanocomposites possess potent antifungal activity against *C. auris*.

The fungal cell wall, a critical structure absent in humans, is a prime target for antifungal therapy. We observed significant alterations in the composition of cell wall of *C. auris* when treated with Ag-*MO* and Ag-Zn-*MO*. Specifically, there was a rise in β-1,3-glucan and chitin, while a decline in mannan levels. This shift in cell wall composition is likely responsible for the enhanced antifungal activity of Ag-*MO* and Ag-Zn-*MO*. *C. auris* typically masks its β-1,3-glucan to evade immune detection. By exposing this crucial component, Ag-*MO* and Ag-Zn-*MO* render the fungus more susceptible to immune recognition and clearance. Furthermore, these cell wall changes may contribute to increased sensitivity to cell wall-perturbing agents like CFW and CR, as well as enhanced cell sedimentation rates. Also, the fungal vacuole, a complex organelle essential for maintaining cellular homeostasis, plays a pivotal role in various cellular processes, including nutrient storage, ion homeostasis, and detoxification. Therefore, we examined the morphology and acidification of the vacuole in the presence of Ag-*MO* and Ag-Zn-*MO* and found that both were significantly impaired. Alterations in membrane permeability are a major mechanism underlying the development of drug resistance. Therefore, understanding the specific mode of action of Ag-*MO* and Ag-Zn-*MO* is crucial to their potential clinical application.

One of the main causes of multidrug resistance in *Candida* is the overexpression of efflux pump. To investigate the impact of Ag-*MO* and Ag-Zn-*MO* on efflux pump function, we observed a significant impairment in efflux pump activity. Interestingly, after phenotypic susceptibility tests conducted with R6G, and NR it was demonstrated that the inhibition was unique to ABC superfamily transporters. Whereas NR is a substrate for both ABC and MFS superfamily transporters, R6G is a particular substrate for ABC superfamily transporters. Furthermore, Lineweaver-Burk plots revealed that both Ag-*MO* and Ag-Zn-*MO* exhibited competitive mode of inhibition. This could be attributed to the preferential localization of ABC transporters in ergosterol-rich regions [42,43]. As our study demonstrated a reduction in ergosterol levels in the presence of both the nanocomposites, this likely contributes to the observed inhibition of ABC transporter activity.

We further investigated the impact of Ag-*MO* and Ag-Zn-*MO* on biofilm formation and found that both nanocomposites effectively inhibited biofilm development. Biofilm formation, initiated by yeast cell adhesion to host surfaces like buccal epithelial cells or denture surfaces, plays an important role in the development of clinical manifestations. Given that biofilm formation begins with cell adhesion, we assessed the impact of Ag-*MO* and Ag-Zn-*MO* on buccal epithelial cell adherence and observed a significant reduction in the presence of these nanocomposites. These findings highlight the potent inhibitory effects of Ag-*MO* and Ag-Zn-*MO* on key virulence traits of *C. auris*.

Lastly, to evaluate the toxicity of our nanocomposites to the host, we performed in-vivo efficacy experiments on two models, THP-1 cell lines *& C. elegans*, and we found that Ag-*MO* and Ag-Zn-*MO* both are of non-toxic nature. Intestinal persistence, which shows less infection in the presence of Ag-*MO* and Ag-Zn-*MO* due to less staining of CFW, further supported their ability to protect the host from *C. auris* infection. Similarly, the course of systemic *C. auris* illness is also known to be determined by the host-pathogen interaction.

Activated macrophages are key intracellular pathogen reservoirs for the host’s defense against invasive fungus. It is known that phagocytosis is a defensive response triggered by activated macrophage-mediated killing [44]. The low CFU in our investigation indicated that destruction of macrophages to facilitate phagocytosis in the presence of Ag-*MO* and Ag-Zn-*MO* was enhanced, indicating that the *C. auris* treated with these nanocomposites had limited infectivity [45]. These findings demonstrate that both our nanocomposites inhibit *C. auris’*s immunological escape pathways.

## 5. Conclusion

The small number of antifungal medications that are currently on the market have drawbacks. Given the urgent situation at hand, current investigation aimed at developing novel biomaterials comprising silver-*MO* and silver-zinc-*MO* nanocomposites that target several sites crucial to immune recognition and *Candida* pathogenicity. This study comprehensively addresses the urgent global health threat posed by *Candida auris*, a notorious multi-drug resistant superbug. By presenting a novel and environmentally conscious approach, this research successfully synthesized silver (Ag-*MO*) and silver-zinc (Ag-Zn-*MO*) nanocomposites utilizing a simple and eco-friendly *Moringa Oleifera* (*MO*) leaf extract-mediated method. The synthesized nanocomposites were thoroughly characterized using a suite of advanced techniques, including FTIR, XRD, TGA, XPS, and SEM, confirming their successful formation and structural integrity. Crucially, the study demonstrated the effective antifungal action of these nanocomposites against *C. auris* in vitro. Mechanistic investigations provided profound insights into how these nanocomposites exert their antifungal effects, revealing a multi-pronged attack on *C. auris*. They were found to alter cell wall composition, impair vital efflux pump activity, induce dysfunctional vacuoles, and significantly suppress biofilm formation – all critical virulence factors contributing to *C. auris*’s persistence and resistance. Furthermore, the therapeutic potential of these nanocomposites was robustly validated through *in vivo* efficacy studies using a nematode model of *Caenorhabditis elegans*.

## Supporting information

S1 File XPS analysis for the prepared Ag NPs. (a) displays the whole XPS scan (survey) for the sample reveling all of its contents. The main goal of this analysis was to check the presence and oxidation state of Ag. (b) shows the Ag 3d peaks splitted over two positions, 367.5 eV for Ag 3d_3/2_ and 373.7 for Ag 3d_5/2_ corresponding to Ag in metallic state (Ag^0^). (c) displays the C 1s XPS spectrum showing broad peak overlapping three positions according to C = O at 288.2 eV, C-O-C at 286.3 eV, and C-C at 284.6 eV. (d) displays the XPS spectrum in the O 1s region which shows an overlapping peak for the O in C-O-C at 533.3 eV, and in C = O at 531.1 eV, supported by C 1s XPS spectrum (Figure S 1 (c)).
(DOCX)

S2 File For the XPS of ZnO/Ag Nano composite. Figures clearly show the phase of Ag, and for Zn spectrum also show peaks confirming the presence also of ZnOH and other Zn oxides derivatives.
(DOCX)

S3 File Growth dynamics of *C. auris.* Figure showing growth dynamics of *C. auris* monitored by measuring optical density at 600 nm of cells grown in absence (control) and presence of Ag-MO (Upper panel) and Ag-Zn-MO (lower panel).
(DOCX)

S4 File Cell viability assay of Ag-*MO* and Ag-Zn-*MO.* Bar graph depicting the % cell viability at MIC of Ag-*MO* and Ag-Zn-*MO*.
(DOCX)

S5 File Effect of Ag-MO and Ag-Zn-*MO* on cell sedimentation of *C. auris.* Lower panel shows the time kinetics of cell sedimentation Ag-*MO* and Ag-Zn-*MO* Treated cells. The upper panel shows the significantly enhanced sedimentation rate of Ag-*MO* and Ag-Zn-*MO* treated cells measuring the difference in absorbance from 0 till 30 min per unit time interval.
(DOCX)

S6 File Antifungal activity of Ag-*MO* and Ag-Zn-*MO.* (DOCX)

S7 File MTT Assay of Ag-*MO* and Ag-Zn-*MO* nanocomposites. (DOCX)

S8 File Haemolytic Percentage of Ag-*MO* and Ag-Zn-*MO* with PBS as negative control and Triton as positive control. (DOCX)

S9 File Static/cidal assay of Ag-*MO* and Ag-Zn-*MO.* (DOCX)

S10 File Ergosterol profiles of *C. auris* scanned between 230 and 300 nm in the absence and presence of Ag-*MO* and Ag-Zn-*MO.* (DOCX)

S11 File Relative percentages of Ergosterol content in absence and presence of Ag-*MO* and Ag-Zn-*MO.* (DOCX)

S12 File Extracellular R6G concentrations for efflux pump mechanism of Ag-*MO.* (DOCX)

S13 File Mode of inhibition via Lineweaver-Burk plot (Ag-*MO*). (DOCX)

S14 File Extracellular R6G concentrations for efflux pump mechanism of Ag-Zn-*MO.* (DOCX)

S15 File Mode of inhibition via Lineweaver-Burk plot (Ag-Zn-*MO*). (DOCX)

S16 File Toxicity of Ag-*MO* and Ag-Zn-*MO* on *C. elegans* showing number of *C. elegans* in the presence of Ag-*MO* and Ag-Zn-*MO* at sub-MIC concentration. (DOCX)

S17 File Percentage survival of *C. auris*–infected *C. elegans* in the presence of Ag-*MO* and Ag-Zn-*MO* until 7 days. (DOCX)

S18 File Macrophage killing assay. Bar graph with CFU log10^5^/ ml on the y-axis after 24-h infection with control (-Ag-*MO* and - Ag-Zn-*MO*) and treated (**+**Ag-*MO* & **+ **Ag-Zn-*MO) C. auris* depicting the killing of fungi by macrophages.
(DOCX)
