# Supplementary material for: Candidacidal effect of Moringa stabilized silver nanomaterials reveal disruption of cell wall integrity, efflux pump, vacuole homeostasis and virulence traits in Candida auris
Source: PLoS One. 2025 Nov 19;20(11):e0336309. doi: 10.1371/journal.pone.0336309 (PMC12629489; doi:10.1371/journal.pone.0336309)
Supplement: S1 File — (a) displays the whole XPS scan (survey) for the sample reveling all of its contents. The main goal of this analysis was to check the presence and oxidation state of Ag. (b) shows the Ag 3d peaks splitted over two positions, 367.5 eV for Ag 3d3/2 and 373.7 for Ag 3d5/2 corresponding to Ag in metallic state (Ag0). (c) displays the C 1s XPS spectrum showing broad peak overlapping three positions according to C = O at 288.2 eV, C-O-C at 286.3 eV, and C-C at 284.6 eV. (d) displays the XPS spectrum in the O 1s region which shows an overlapping peak for the O in C-O-C at 533.3 eV, and in C = O at 531.1 eV, supported by C 1s XPS spectrum (Figure S 1 (c)). (DOCX) [file pone.0336309.s001.docx]

1. **XPS of Ag-*MO* and Ag-Zn-*MO***

**S1 File. XPS analysis for the prepared Ag NPs.**


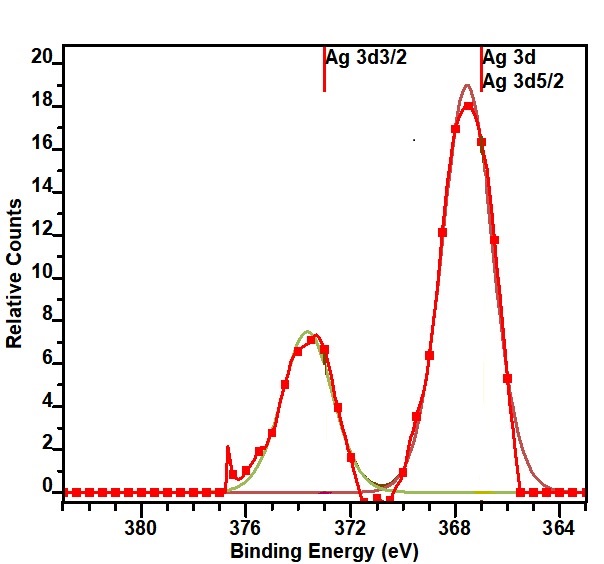

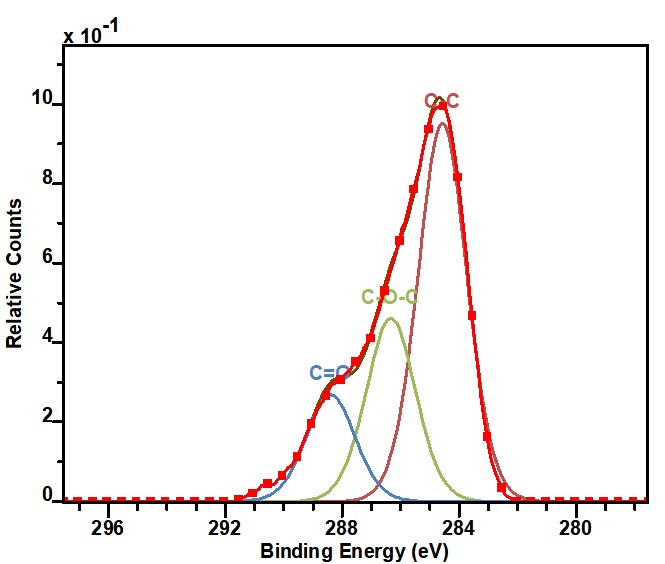

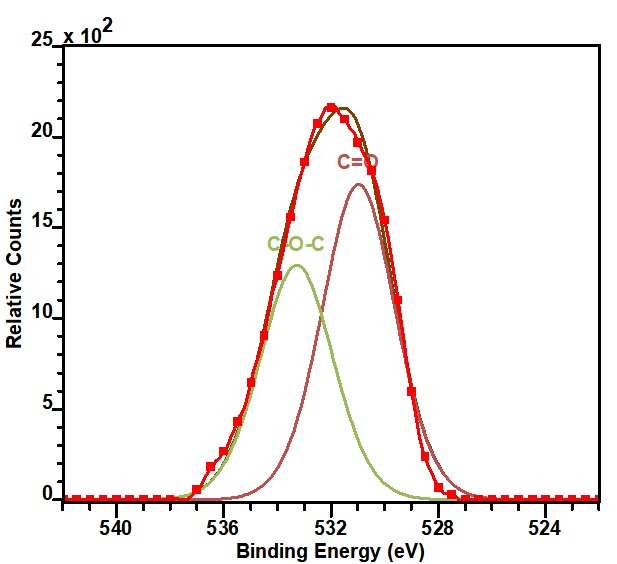

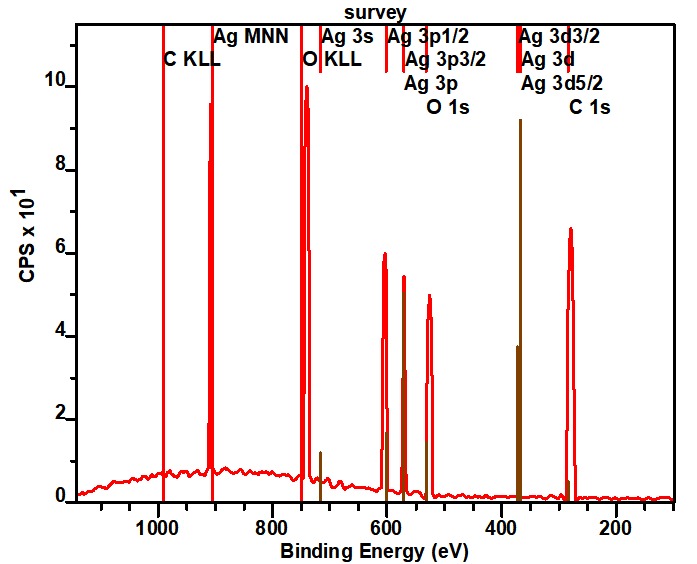


**C 1S**

**O 1S**

**(d)**

**(c)**

**(b)**

**(a)**

XPS analysis was done to confirm the formation of Ag NPs presuming the elements and oxidation states in the sample. S 1 (a) displays the whole XPS scan (survey) for the sample reveling all of its contents. The main goal of this analysis was to check the presence and oxidation state of Ag. S 1 (b) shows the Ag 3d peaks splitted over two positions, 367.5 eV for Ag 3d_3/2_ and 373.7 for Ag 3d_5/2_ corresponding to Ag in metallic state (Ag^0^). Figure S 1 (C) displays the C 1s XPS spectrum showing broad peak overlapping three positions according to C=O at 288.2 eV, C-O-C at 286.3 eV, and C-C at 284.6 eV.

These carbon derivatives came from the residual of Moringa extract used in the preparation of the Ag NPs. S 1 (d) displays the XPS spectrum in the O 1s region which shows an overlapping peak for the O in C-O-C at 533.3 eV, and in C=O at 531.1 eV, supported by C 1s XPS spectrum (S 1 (C).

The XPS results confirm the formation of Metallic Ag NPs without any presence of AgO.
