# Supplementary material for: Candidacidal effect of Moringa stabilized silver nanomaterials reveal disruption of cell wall integrity, efflux pump, vacuole homeostasis and virulence traits in Candida auris
Source: PLoS One. 2025 Nov 19;20(11):e0336309. doi: 10.1371/journal.pone.0336309 (PMC12629489; doi:10.1371/journal.pone.0336309)
Supplement: S2 File — Figures clearly show the phase of Ag, and for Zn spectrum also show peaks confirming the presence also of ZnOH and other Zn oxides derivatives. (DOCX) [file pone.0336309.s002.docx]

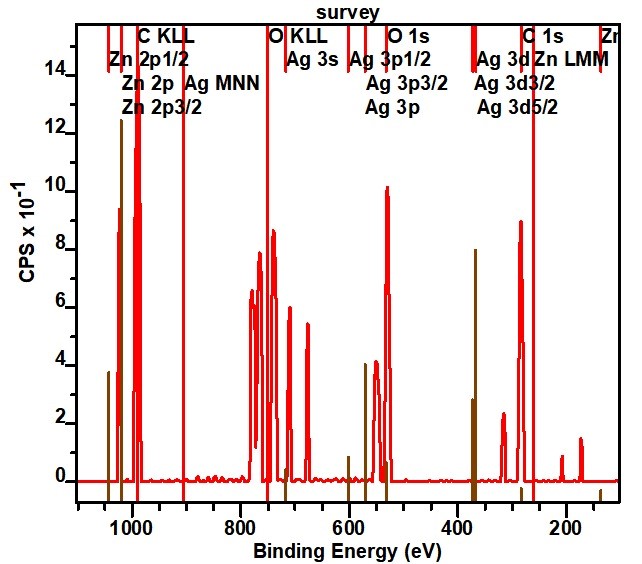

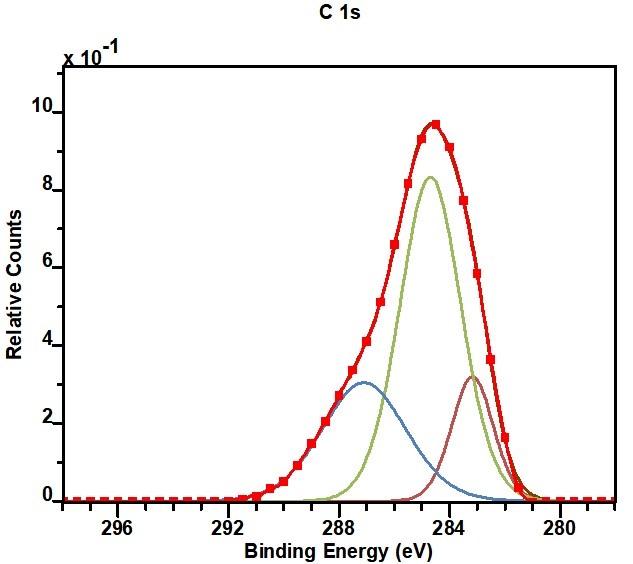

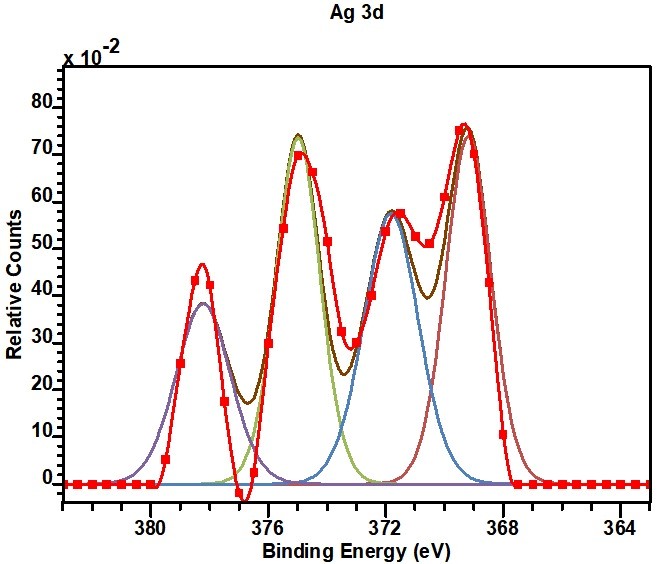

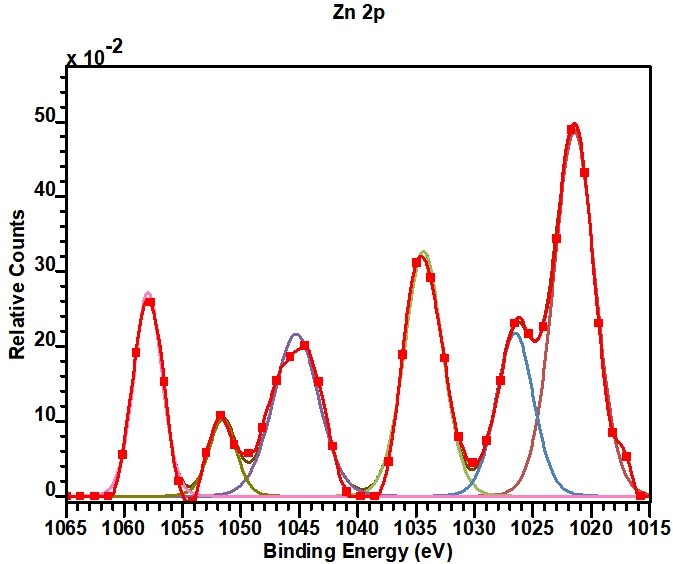


**S2 File**: **XPS of ZnO/Ag nano composite.**

XPS analysis was done to confirm the formation of Ag/Zn NPs presuming the elements and oxidation states in the sample. Figures clearly show the phase of Ag, and for Zn spectrum also show peaks confirming the presence also of ZnOH and other Zn oxides derivatives.
