# Supplementary material for: Candidacidal effect of Moringa stabilized silver nanomaterials reveal disruption of cell wall integrity, efflux pump, vacuole homeostasis and virulence traits in Candida auris
Source: PLoS One. 2025 Nov 19;20(11):e0336309. doi: 10.1371/journal.pone.0336309 (PMC12629489; doi:10.1371/journal.pone.0336309)
Supplement: S3 File — Figure showing growth dynamics of C. auris monitored by measuring optical density at 600 nm of cells grown in absence (control) and presence of Ag-MO (Upper panel) and Ag-Zn-MO (lower panel). (DOCX) [file pone.0336309.s003.docx]

**S 3 File showing growth dynamics of *C. auris* monitored by measuring optical density at 600 nm of cells grown in absence (control) and presence of Ag-*MO* (Upper panel) and Ag-Zn-*MO* (lower panel)**
