# Supplementary material for: Candidacidal effect of Moringa stabilized silver nanomaterials reveal disruption of cell wall integrity, efflux pump, vacuole homeostasis and virulence traits in Candida auris
Source: PLoS One. 2025 Nov 19;20(11):e0336309. doi: 10.1371/journal.pone.0336309 (PMC12629489; doi:10.1371/journal.pone.0336309)
Supplement: S4 File — Bar graph depicting the % cell viability at MIC of Ag-MO and Ag-Zn-MO. (DOCX) [file pone.0336309.s004.docx]

**S 4 File. Cell viability assay of Ag-*MO* and Ag-Zn-*MO*. Bar graph depicting the % cell viability at MIC of Ag-*MO* and Ag-Zn-*MO*.**
