# Supplementary material for: Candidacidal effect of Moringa stabilized silver nanomaterials reveal disruption of cell wall integrity, efflux pump, vacuole homeostasis and virulence traits in Candida auris
Source: PLoS One. 2025 Nov 19;20(11):e0336309. doi: 10.1371/journal.pone.0336309 (PMC12629489; doi:10.1371/journal.pone.0336309)
Supplement: S5 File — Lower panel shows the time kinetics of cell sedimentation Ag-MO and Ag-Zn-MO Treated cells. The upper panel shows the significantly enhanced sedimentation rate of Ag-MO and Ag-Zn-MO treated cells measuring the difference in absorbance from 0 till 30 min per unit time interval. (DOCX) [file pone.0336309.s005.docx]

**S 5 File: Effect of Ag-*MO* and Ag-Zn-*MO* on cell sedimentation of *C. auris*. Lower panel shows the time kinetics of cell sedimentation Ag-*MO* and Ag-Zn-*MO* Treated cells. The upper panel shows the significantly enhanced sedimentation rate of Ag-*MO* and Ag-Zn-*MO* treated cells measuring the difference in absorbance from 0 till 30 min per unit time interval**
