# Supplementary material for: Candidacidal effect of Moringa stabilized silver nanomaterials reveal disruption of cell wall integrity, efflux pump, vacuole homeostasis and virulence traits in Candida auris
Source: PLoS One. 2025 Nov 19;20(11):e0336309. doi: 10.1371/journal.pone.0336309 (PMC12629489; doi:10.1371/journal.pone.0336309)
Supplement: S6 File — (DOCX) [file pone.0336309.s006.docx]

**S6 Fig. Antifungal activity of Ag-*MO* and Ag-Zn-*MO*.**

| **Concentrations** | **Ag-*MO*** | **Ag-Zn-*MO*** |
| --- | --- | --- |
| 2.00 µg/mL | 1.353 | 1.446 |
| 4.01 µg/mL | 1.353 | 1.442 |
| 8.03 µg/mL | 1.374 | 1.468 |
| 16.05 µg/mL | 1.36 | 1.42 |
| 32.15 µg/mL | 1.21 | 1.41 |
| 62.5 µg/mL | 0.81 | 1.378 |
| 125 µg/mL | 0.08 | 1.3053 |
| 250 µg/mL | 0.093 | 0.1157 |
| 500 µg/mL | 0.22 | 0.084 |

**S7 Fig.** **MTT Assay of Ag-*MO* and Ag-Zn-*MO* nanocomposites.**

|  | **Control** | **Ag-*MO***  **(100 µg/mL)** | **Ag-Zn-*MO***  **(200 µg/mL)** |
| --- | --- | --- | --- |
| **Biomass** | 3.7 | 1.3 | 1.8 |
| **Biofilm** | 3.5 | 1.28 | 1.1 |

**S8 Fig Haemolytic Percentage of Ag-*MO* and Ag-Zn-*MO* with PBS as negative control and Triton as positive control.**

| **Sample** | **Haemolytic %** |
| --- | --- |
| PBS | 0 |
| TRITON | 100 |
| Ag-MO (MIC) | 3 |
| Ag-MO (2X MIC)) | 9 |
| Ag-Zn-MO(MIC) | 3 |
| Ag-Zn-MO (2X MIC) | 12 |

**S9 Fig. Static/cidal assay of Ag-*MO* and Ag-Zn-*MO***

|  | **Absorbance on DAY 1 (nm)** | **Absorbance on DAY 2 (nm)** |
| --- | --- | --- |
| **Control** | 10.43 | 8.68 |
| **Ag-MO** | 1.68 | 0.75 |
| **Ag-Zn-MO** | 2.47 | 8.35 |

**S10 Fig. Ergosterol profiles of *C. auris* scanned between 230 and 300 nm in the absence and presence of Ag-*MO* and Ag-Zn-*MO***

| **Absorbance (nm)** | **Control** | **Wavelength (nm)**  **Ag-*MO*** | **Wavelength (nm)**  **Ag-Zn-*MO*** |
| --- | --- | --- | --- |
| 230 | 0.031 | 0.014 | 0.0248 |
| 230.5 | 0.03 | 0.015 | 0.024 |
| 231 | 0.028 | 0.012 | 0.0224 |
| 231.5 | 0.031 | 0.011 | 0.0248 |
| 232 | 0.029 | 0.011 | 0.0232 |
| 232.5 | 0.028 | 0.011 | 0.0224 |
| 233 | 0.029 | 0.012 | 0.0232 |
| 233.5 | 0.029 | 0.011 | 0.0232 |
| 234 | 0.027 | 0.009 | 0.0216 |
| 234.5 | 0.024 | 0.009 | 0.0192 |
| 235 | 0.024 | 0.01 | 0.0192 |
| 235.5 | 0.024 | 0.008 | 0.0192 |
| 236 | 0.024 | 0.008 | 0.0192 |
| 236.5 | 0.025 | 0.008 | 0.02 |
| 237 | 0.026 | 0.009 | 0.0208 |
| 237.5 | 0.026 | 0.01 | 0.0208 |
| 238 | 0.025 | 0.009 | 0.02 |
| 238.5 | 0.025 | 0.008 | 0.02 |
| 239 | 0.025 | 0.008 | 0.02 |
| 239.5 | 0.026 | 0.009 | 0.0208 |
| 240 | 0.024 | 0.009 | 0.0192 |
| 240.5 | 0.025 | 0.009 | 0.02 |
| 241 | 0.027 | 0.011 | 0.0216 |
| 241.5 | 0.028 | 0.013 | 0.0224 |
| 242 | 0.027 | 0.013 | 0.0216 |
| 242.5 | 0.028 | 0.013 | 0.0224 |
| 243 | 0.03 | 0.012 | 0.024 |
| 243.5 | 0.032 | 0.015 | 0.0256 |
| 244 | 0.032 | 0.015 | 0.0256 |
| 244.5 | 0.032 | 0.014 | 0.0256 |
| 245 | 0.031 | 0.016 | 0.0248 |
| 245.5 | 0.032 | 0.016 | 0.0256 |
| 246 | 0.032 | 0.018 | 0.0256 |
| 246.5 | 0.034 | 0.019 | 0.0272 |
| 247 | 0.034 | 0.019 | 0.0272 |
| 247.5 | 0.037 | 0.02 | 0.0296 |
| 248 | 0.039 | 0.022 | 0.0312 |
| 248.5 | 0.039 | 0.024 | 0.0312 |
| 249 | 0.04 | 0.024 | 0.032 |
| 249.5 | 0.043 | 0.025 | 0.0344 |
| 250 | 0.045 | 0.027 | 0.036 |
| 250.5 | 0.047 | 0.028 | 0.0376 |
| 251 | 0.048 | 0.028 | 0.0384 |
| 251.5 | 0.047 | 0.031 | 0.0376 |
| 252 | 0.051 | 0.03 | 0.0408 |
| 252.5 | 0.051 | 0.032 | 0.0408 |
| 253 | 0.05 | 0.031 | 0.04 |
| 253.5 | 0.051 | 0.034 | 0.0408 |
| 254 | 0.053 | 0.033 | 0.0424 |
| 254.5 | 0.054 | 0.036 | 0.0432 |
| 255 | 0.056 | 0.035 | 0.0448 |
| 255.5 | 0.057 | 0.036 | 0.0456 |
| 256 | 0.058 | 0.038 | 0.0464 |
| 256.5 | 0.06 | 0.039 | 0.048 |
| 257 | 0.061 | 0.041 | 0.0488 |
| 257.5 | 0.063 | 0.044 | 0.0504 |
| 258 | 0.066 | 0.043 | 0.0528 |
| 258.5 | 0.07 | 0.048 | 0.056 |
| 259 | 0.07 | 0.05 | 0.056 |
| 259.5 | 0.072 | 0.051 | 0.0576 |
| 260 | 0.076 | 0.055 | 0.0608 |
| 260.5 | 0.075 | 0.054 | 0.06 |
| 261 | 0.075 | 0.053 | 0.06 |
| 261.5 | 0.079 | 0.055 | 0.0632 |
| 262 | 0.078 | 0.055 | 0.0624 |
| 262.5 | 0.077 | 0.056 | 0.0616 |
| 263 | 0.077 | 0.054 | 0.0616 |
| 263.5 | 0.078 | 0.055 | 0.0624 |
| 264 | 0.077 | 0.058 | 0.0616 |
| 264.5 | 0.08 | 0.059 | 0.064 |
| 265 | 0.079 | 0.058 | 0.0632 |
| 265.5 | 0.081 | 0.061 | 0.0648 |
| 266 | 0.081 | 0.063 | 0.0648 |
| 266.5 | 0.083 | 0.064 | 0.0664 |
| 267 | 0.087 | 0.067 | 0.0696 |
| 267.5 | 0.088 | 0.068 | 0.0704 |
| 268 | 0.09 | 0.07 | 0.072 |
| 268.5 | 0.094 | 0.071 | 0.0752 |
| 269 | 0.099 | 0.077 | 0.0792 |
| 269.5 | 0.102 | 0.076 | 0.0816 |
| 270 | 0.104 | 0.077 | 0.0832 |
| 270.5 | 0.103 | 0.079 | 0.0824 |
| 271 | 0.105 | 0.082 | 0.084 |
| 271.5 | 0.105 | 0.082 | 0.084 |
| 272 | 0.104 | 0.081 | 0.0832 |
| 272.5 | 0.099 | 0.079 | 0.0792 |
| 273 | 0.097 | 0.078 | 0.0776 |
| 273.5 | 0.096 | 0.075 | 0.0768 |
| 274 | 0.094 | 0.07 | 0.0752 |
| 274.5 | 0.091 | 0.07 | 0.0728 |
| 275 | 0.088 | 0.07 | 0.0704 |
| 275.5 | 0.088 | 0.069 | 0.0704 |
| 276 | 0.09 | 0.069 | 0.072 |
| 276.5 | 0.089 | 0.065 | 0.0712 |
| 277 | 0.091 | 0.071 | 0.0728 |
| 277.5 | 0.091 | 0.071 | 0.0728 |
| 278 | 0.091 | 0.07 | 0.0728 |
| 278.5 | 0.092 | 0.07 | 0.0736 |
| 279 | 0.098 | 0.076 | 0.0784 |
| 279.5 | 0.103 | 0.077 | 0.0824 |
| 280 | 0.106 | 0.08 | 0.0848 |
| 280.5 | 0.106 | 0.081 | 0.0848 |
| 281 | 0.107 | 0.084 | 0.0856 |
| 281.5 | 0.108 | 0.084 | 0.0864 |
| 282 | 0.105 | 0.081 | 0.084 |
| 282.5 | 0.103 | 0.079 | 0.0824 |
| 283 | 0.1 | 0.078 | 0.08 |
| 283.5 | 0.096 | 0.075 | 0.0768 |
| 284 | 0.093 | 0.07 | 0.0744 |
| 284.5 | 0.088 | 0.065 | 0.0704 |
| 285 | 0.082 | 0.062 | 0.0656 |
| 285.5 | 0.077 | 0.055 | 0.0616 |
| 286 | 0.07 | 0.053 | 0.056 |
| 286.5 | 0.065 | 0.05 | 0.052 |
| 287 | 0.06 | 0.045 | 0.048 |
| 287.5 | 0.06 | 0.042 | 0.048 |
| 288 | 0.054 | 0.037 | 0.0432 |
| 288.5 | 0.053 | 0.038 | 0.0424 |
| 289 | 0.053 | 0.038 | 0.0424 |
| 289.5 | 0.053 | 0.037 | 0.0424 |
| 290 | 0.055 | 0.036 | 0.044 |
| 290.5 | 0.052 | 0.035 | 0.0416 |
| 291 | 0.052 | 0.035 | 0.0416 |
| 291.5 | 0.053 | 0.038 | 0.0424 |
| 292 | 0.054 | 0.037 | 0.0432 |
| 292.5 | 0.056 | 0.038 | 0.0448 |
| 293 | 0.058 | 0.041 | 0.0464 |
| 293.5 | 0.059 | 0.043 | 0.0472 |
| 294 | 0.056 | 0.04 | 0.0448 |
| 294.5 | 0.057 | 0.037 | 0.0456 |
| 295 | 0.055 | 0.035 | 0.044 |
| 295.5 | 0.051 | 0.034 | 0.0408 |
| 296 | 0.048 | 0.031 | 0.0384 |
| 296.5 | 0.042 | 0.029 | 0.0336 |
| 297 | 0.041 | 0.026 | 0.0328 |
| 297.5 | 0.034 | 0.022 | 0.0272 |
| 298 | 0.03 | 0.015 | 0.024 |
| 298.5 | 0.025 | 0.011 | 0.02 |
| 299 | 0.019 | 0.005 | 0.0152 |
| 299.5 | 0.015 | 0.004 | 0.012 |
| 300 | 0.016 | 0.004 | 0.0128 |

**S11 Fig. Relative percentages of Ergosterol content in absence and presence of Ag-*MO* and Ag-Zn-*MO*.**

| **Sample** | **E1** | **E2** | **E3** | **Mean** | **SD** |
| --- | --- | --- | --- | --- | --- |
| **Control** | 0.0063 | 0.0064 | 0.0062 | 0.0063 | 0.0001 |
| **Ag-MO** | 0.002 | 0.003 | 0.002 | 0.002 | 0.0005 |
| **Ag-Zn-MO** | 0.001 | 0.002 | 0.002 | 0.001 | 0.0005 |

**S12 Fig. Extracellular R6G concentrations for efflux pump mechanism of Ag-*MO***

| **Time** | **Positive Control** | **Ag-*MO*** | **Negative Control** |
| --- | --- | --- | --- |
| 0 min | 0.102 | 0.103 | 0.101 |
| 10 min | 0.184 | 0.178 | 0.104 |
| 20 min | 0.227 | 0.188 | 0.107 |
| 30 min | 0.229 | 0.198 | 0.106 |
| 40 min | 0.233 | 0.201 | 0.111 |
| 50 min | 0.236 | 0.217 | 0.122 |
| 60 min | 0.239 | 0.218 | 0.103 |
| 70 min | 0.246 | 0.224 | 0.123 |
| 80 min | 0.263 | 0.226 | 0.121 |
| 90 min | 0.274 | 0.232 | 0.124 |

**S13 Fig. Mode of inhibition via Lineweaver-Burk plot (Ag-*MO*)**

| **Time** | **1/s** | **1/v treated** | **1/v control** |
| --- | --- | --- | --- |
| 40 | 0.025 | 11.12 | 7.91 |
| 30 | 0.033333 | 11.71 | 7.33 |
| 20 | 0.05 | 11.89 | 8.41 |
| 10 | 0.1 | 14.92 | 9.77 |

**S14 Fig. Extracellular R6G concentrations for efflux pump mechanism of Ag-Zn-*MO***

| **Time** | **Positive Control** | **Ag-Zn-*MO*** | **Negative Control** |
| --- | --- | --- | --- |
| 0 min | 0.087 | 0.085 | 0.086 |
| 10 min | 0.112 | 0.097 | 0.086 |
| 20 min | 0.116 | 0.098 | 0.085 |
| 30 min | 0.119 | 0.101 | 0.084 |
| 40 min | 0.124 | 0.104 | 0.086 |
| 50 min | 0.13 | 0.109 | 0.085 |
| 60 min | 0.154 | 0.113 | 0.087 |
| 70 min | 0.201 | 0.135 | 0.089 |
| 80 min | 0.208 | 0.14 | 0.09 |
| 90 min | 0.214 | 0.143 | 0.091 |

**S15 Fig. Mode of inhibition via Lineweaver-Burk plot (Ag-Zn-*MO*)**

| **Time** | **1/s** | **1/v Treated** | **1/v Control** |
| --- | --- | --- | --- |
| 40 | 0.021 | 16.97 | 18.9 |
| 30 | 0.033 | 23.09 | 21.32 |
| 20 | 0.05 | 34.96 | 26.52 |
| 10 | 0.1 | 38.75 | 51.81 |

**S16 Fig. Toxicity of Ag-*MO* and Ag-Zn-*MO* on *C. elegans* showing number of *C. elegans* in the presence of Ag-*MO* and Ag-Zn-*MO* at sub-MIC concentration**

| **Day** | **Control**  **(number of *C.elegans*)** | **Ag-*MO***  **(number of *C.elegans*)** | **Ag-Zn-*MO***  **(number of *C.elegans*)** |
| --- | --- | --- | --- |
| 0.99 | 50 | 50 | 50 |
| 1 | 50 | 50 | 50 |
| 1.99 | 50 | 46 | 47 |
| 2 | 50 | 46 | 47 |
| 2.99 | 50 | 46 | 47 |
| 3 | 48 | 42 | 47 |
| 3.99 | 48 | 42 | 44 |
| 4 | 48 | 42 | 41 |
| 4.99 | 48 | 40 | 41 |
| 5 | 48 | 40 | 41 |
| 5.99 | 46 | 36 | 38 |
| 6 | 46 | 36 | 38 |
| 6.99 | 44 | 36 | 38 |
| 7 | 44 | 36 | 35 |

**S17 Fig. Percentage survival of *C. auris*–infected *C. elegans* in the presence of Ag-*MO* and Ag-Zn-*MO* until 7 days.**

| **Day** | **Control**  **(Number of *C.elegans*)** | **Ag-MO**  **(Number of *C.elegans*)** | **Ag-Zn-MO**  **(Number of *C.elegans*)** |
| --- | --- | --- | --- |
| 0.99 | 50 | 50 | 50 |
| 1 | 45 | 50 | 50 |
| 1.99 | 45 | 50 | 50 |
| 2 | 45 | 50 | 47 |
| 2.99 | 42 | 48 | 47 |
| 3 | 42 | 48 | 47 |
| 3.99 | 38 | 48 | 44 |
| 4 | 38 | 48 | 44 |
| 4.99 | 38 | 47 | 44 |
| 5 | 34 | 47 | 41 |
| 5.99 | 34 | 45 | 41 |
| 6 | 34 | 45 | 41 |
| 6.99 | 30 | 45 | 40 |
| 7 | 30 | 43 | 40 |

**S18 Fig. Macrophage killing assay. Bar graph with CFU log10^5^/ ml on the y-axis after 24-h infection with control (-Ag-*MO* and - Ag-Zn-*MO*) and treated (+Ag-*MO* & +Ag-Zn-*MO) C. auris* depicting the killing of fungi by macrophages.**

| **Sample** | **(10^5^ CFU)**  **E1** | **(10^5^ CFU)**  **E2** | **(10^5^ CFU)**  **E3** | **Mean** | **Standard Deviation** |
| --- | --- | --- | --- | --- | --- |
| Control | 138.66 | 137.24 | 140.074 | 138.65 | 1.42 |
| Ag-MO | 46 | 44.58 | 47.41 | 45.99 | 1.41 |
| Ag-Zn-MO | 55.66 | 54.24 | 57.07 | 55.66 | 1.42 |
