# Supplementary material for: Candidacidal effect of Moringa stabilized silver nanomaterials reveal disruption of cell wall integrity, efflux pump, vacuole homeostasis and virulence traits in Candida auris
Source: PLoS One. 2025 Nov 19;20(11):e0336309. doi: 10.1371/journal.pone.0336309 (PMC12629489; doi:10.1371/journal.pone.0336309)
Supplement: S7 File — (DOCX) [file pone.0336309.s007.docx]

**S7 File.** **MTT Assay of Ag-*MO* and Ag-Zn-*MO* nanocomposites.**

|  | **Control** | **Ag-*MO***  **(100 µg/mL)** | **Ag-Zn-*MO***  **(200 µg/mL)** |
| --- | --- | --- | --- |
| **Biomass** | 3.7 | 1.3 | 1.8 |
| **Biofilm** | 3.5 | 1.28 | 1.1 |
