# Supplementary material for: Candidacidal effect of Moringa stabilized silver nanomaterials reveal disruption of cell wall integrity, efflux pump, vacuole homeostasis and virulence traits in Candida auris
Source: PLoS One. 2025 Nov 19;20(11):e0336309. doi: 10.1371/journal.pone.0336309 (PMC12629489; doi:10.1371/journal.pone.0336309)
Supplement: S8 File — (DOCX) [file pone.0336309.s008.docx]

**S8 File Haemolytic Percentage of Ag-*MO* and Ag-Zn-*MO* with PBS as negative control and Triton as positive control.**

| **Sample** | **Haemolytic %** |
| --- | --- |
| PBS | 0 |
| TRITON | 100 |
| Ag-MO (MIC) | 3 |
| Ag-MO (2X MIC)) | 9 |
| Ag-Zn-MO(MIC) | 3 |
| Ag-Zn-MO (2X MIC) | 12 |
