# Supplementary material for: Candidacidal effect of Moringa stabilized silver nanomaterials reveal disruption of cell wall integrity, efflux pump, vacuole homeostasis and virulence traits in Candida auris
Source: PLoS One. 2025 Nov 19;20(11):e0336309. doi: 10.1371/journal.pone.0336309 (PMC12629489; doi:10.1371/journal.pone.0336309)
Supplement: S9 File — (DOCX) [file pone.0336309.s009.docx]

**S9 File. Static/cidal assay of Ag-*MO* and Ag-Zn-*MO***

|  | **Absorbance on DAY 1 (nm)** | **Absorbance on DAY 2 (nm)** |
| --- | --- | --- |
| **Control** | 10.43 | 8.68 |
| **Ag-MO** | 1.68 | 0.75 |
| **Ag-Zn-MO** | 2.47 | 8.35 |
