# Supplementary material for: Candidacidal effect of Moringa stabilized silver nanomaterials reveal disruption of cell wall integrity, efflux pump, vacuole homeostasis and virulence traits in Candida auris
Source: PLoS One. 2025 Nov 19;20(11):e0336309. doi: 10.1371/journal.pone.0336309 (PMC12629489; doi:10.1371/journal.pone.0336309)
Supplement: S10 File — (DOCX) [file pone.0336309.s010.docx]

**S10 File. Ergosterol profiles of *C. auris* scanned between 230 and 300 nm in the absence and presence of Ag-*MO* and Ag-Zn-*MO***

| **Absorbance (nm)** | **Control** | **Wavelength (nm)**  **Ag-*MO*** | **Wavelength (nm)**  **Ag-Zn-*MO*** |
| --- | --- | --- | --- |
| 230 | 0.031 | 0.014 | 0.0248 |
| 230.5 | 0.03 | 0.015 | 0.024 |
| 231 | 0.028 | 0.012 | 0.0224 |
| 231.5 | 0.031 | 0.011 | 0.0248 |
| 232 | 0.029 | 0.011 | 0.0232 |
| 232.5 | 0.028 | 0.011 | 0.0224 |
| 233 | 0.029 | 0.012 | 0.0232 |
| 233.5 | 0.029 | 0.011 | 0.0232 |
| 234 | 0.027 | 0.009 | 0.0216 |
| 234.5 | 0.024 | 0.009 | 0.0192 |
| 235 | 0.024 | 0.01 | 0.0192 |
| 235.5 | 0.024 | 0.008 | 0.0192 |
| 236 | 0.024 | 0.008 | 0.0192 |
| 236.5 | 0.025 | 0.008 | 0.02 |
| 237 | 0.026 | 0.009 | 0.0208 |
| 237.5 | 0.026 | 0.01 | 0.0208 |
| 238 | 0.025 | 0.009 | 0.02 |
| 238.5 | 0.025 | 0.008 | 0.02 |
| 239 | 0.025 | 0.008 | 0.02 |
| 239.5 | 0.026 | 0.009 | 0.0208 |
| 240 | 0.024 | 0.009 | 0.0192 |
| 240.5 | 0.025 | 0.009 | 0.02 |
| 241 | 0.027 | 0.011 | 0.0216 |
| 241.5 | 0.028 | 0.013 | 0.0224 |
| 242 | 0.027 | 0.013 | 0.0216 |
| 242.5 | 0.028 | 0.013 | 0.0224 |
| 243 | 0.03 | 0.012 | 0.024 |
| 243.5 | 0.032 | 0.015 | 0.0256 |
| 244 | 0.032 | 0.015 | 0.0256 |
| 244.5 | 0.032 | 0.014 | 0.0256 |
| 245 | 0.031 | 0.016 | 0.0248 |
| 245.5 | 0.032 | 0.016 | 0.0256 |
| 246 | 0.032 | 0.018 | 0.0256 |
| 246.5 | 0.034 | 0.019 | 0.0272 |
| 247 | 0.034 | 0.019 | 0.0272 |
| 247.5 | 0.037 | 0.02 | 0.0296 |
| 248 | 0.039 | 0.022 | 0.0312 |
| 248.5 | 0.039 | 0.024 | 0.0312 |
| 249 | 0.04 | 0.024 | 0.032 |
| 249.5 | 0.043 | 0.025 | 0.0344 |
| 250 | 0.045 | 0.027 | 0.036 |
| 250.5 | 0.047 | 0.028 | 0.0376 |
| 251 | 0.048 | 0.028 | 0.0384 |
| 251.5 | 0.047 | 0.031 | 0.0376 |
| 252 | 0.051 | 0.03 | 0.0408 |
| 252.5 | 0.051 | 0.032 | 0.0408 |
| 253 | 0.05 | 0.031 | 0.04 |
| 253.5 | 0.051 | 0.034 | 0.0408 |
| 254 | 0.053 | 0.033 | 0.0424 |
| 254.5 | 0.054 | 0.036 | 0.0432 |
| 255 | 0.056 | 0.035 | 0.0448 |
| 255.5 | 0.057 | 0.036 | 0.0456 |
| 256 | 0.058 | 0.038 | 0.0464 |
| 256.5 | 0.06 | 0.039 | 0.048 |
| 257 | 0.061 | 0.041 | 0.0488 |
| 257.5 | 0.063 | 0.044 | 0.0504 |
| 258 | 0.066 | 0.043 | 0.0528 |
| 258.5 | 0.07 | 0.048 | 0.056 |
| 259 | 0.07 | 0.05 | 0.056 |
| 259.5 | 0.072 | 0.051 | 0.0576 |
| 260 | 0.076 | 0.055 | 0.0608 |
| 260.5 | 0.075 | 0.054 | 0.06 |
| 261 | 0.075 | 0.053 | 0.06 |
| 261.5 | 0.079 | 0.055 | 0.0632 |
| 262 | 0.078 | 0.055 | 0.0624 |
| 262.5 | 0.077 | 0.056 | 0.0616 |
| 263 | 0.077 | 0.054 | 0.0616 |
| 263.5 | 0.078 | 0.055 | 0.0624 |
| 264 | 0.077 | 0.058 | 0.0616 |
| 264.5 | 0.08 | 0.059 | 0.064 |
| 265 | 0.079 | 0.058 | 0.0632 |
| 265.5 | 0.081 | 0.061 | 0.0648 |
| 266 | 0.081 | 0.063 | 0.0648 |
| 266.5 | 0.083 | 0.064 | 0.0664 |
| 267 | 0.087 | 0.067 | 0.0696 |
| 267.5 | 0.088 | 0.068 | 0.0704 |
| 268 | 0.09 | 0.07 | 0.072 |
| 268.5 | 0.094 | 0.071 | 0.0752 |
| 269 | 0.099 | 0.077 | 0.0792 |
| 269.5 | 0.102 | 0.076 | 0.0816 |
| 270 | 0.104 | 0.077 | 0.0832 |
| 270.5 | 0.103 | 0.079 | 0.0824 |
| 271 | 0.105 | 0.082 | 0.084 |
| 271.5 | 0.105 | 0.082 | 0.084 |
| 272 | 0.104 | 0.081 | 0.0832 |
| 272.5 | 0.099 | 0.079 | 0.0792 |
| 273 | 0.097 | 0.078 | 0.0776 |
| 273.5 | 0.096 | 0.075 | 0.0768 |
| 274 | 0.094 | 0.07 | 0.0752 |
| 274.5 | 0.091 | 0.07 | 0.0728 |
| 275 | 0.088 | 0.07 | 0.0704 |
| 275.5 | 0.088 | 0.069 | 0.0704 |
| 276 | 0.09 | 0.069 | 0.072 |
| 276.5 | 0.089 | 0.065 | 0.0712 |
| 277 | 0.091 | 0.071 | 0.0728 |
| 277.5 | 0.091 | 0.071 | 0.0728 |
| 278 | 0.091 | 0.07 | 0.0728 |
| 278.5 | 0.092 | 0.07 | 0.0736 |
| 279 | 0.098 | 0.076 | 0.0784 |
| 279.5 | 0.103 | 0.077 | 0.0824 |
| 280 | 0.106 | 0.08 | 0.0848 |
| 280.5 | 0.106 | 0.081 | 0.0848 |
| 281 | 0.107 | 0.084 | 0.0856 |
| 281.5 | 0.108 | 0.084 | 0.0864 |
| 282 | 0.105 | 0.081 | 0.084 |
| 282.5 | 0.103 | 0.079 | 0.0824 |
| 283 | 0.1 | 0.078 | 0.08 |
| 283.5 | 0.096 | 0.075 | 0.0768 |
| 284 | 0.093 | 0.07 | 0.0744 |
| 284.5 | 0.088 | 0.065 | 0.0704 |
| 285 | 0.082 | 0.062 | 0.0656 |
| 285.5 | 0.077 | 0.055 | 0.0616 |
| 286 | 0.07 | 0.053 | 0.056 |
| 286.5 | 0.065 | 0.05 | 0.052 |
| 287 | 0.06 | 0.045 | 0.048 |
| 287.5 | 0.06 | 0.042 | 0.048 |
| 288 | 0.054 | 0.037 | 0.0432 |
| 288.5 | 0.053 | 0.038 | 0.0424 |
| 289 | 0.053 | 0.038 | 0.0424 |
| 289.5 | 0.053 | 0.037 | 0.0424 |
| 290 | 0.055 | 0.036 | 0.044 |
| 290.5 | 0.052 | 0.035 | 0.0416 |
| 291 | 0.052 | 0.035 | 0.0416 |
| 291.5 | 0.053 | 0.038 | 0.0424 |
| 292 | 0.054 | 0.037 | 0.0432 |
| 292.5 | 0.056 | 0.038 | 0.0448 |
| 293 | 0.058 | 0.041 | 0.0464 |
| 293.5 | 0.059 | 0.043 | 0.0472 |
| 294 | 0.056 | 0.04 | 0.0448 |
| 294.5 | 0.057 | 0.037 | 0.0456 |
| 295 | 0.055 | 0.035 | 0.044 |
| 295.5 | 0.051 | 0.034 | 0.0408 |
| 296 | 0.048 | 0.031 | 0.0384 |
| 296.5 | 0.042 | 0.029 | 0.0336 |
| 297 | 0.041 | 0.026 | 0.0328 |
| 297.5 | 0.034 | 0.022 | 0.0272 |
| 298 | 0.03 | 0.015 | 0.024 |
| 298.5 | 0.025 | 0.011 | 0.02 |
| 299 | 0.019 | 0.005 | 0.0152 |
| 299.5 | 0.015 | 0.004 | 0.012 |
| 300 | 0.016 | 0.004 | 0.0128 |
