# Supplementary material for: Candidacidal effect of Moringa stabilized silver nanomaterials reveal disruption of cell wall integrity, efflux pump, vacuole homeostasis and virulence traits in Candida auris
Source: PLoS One. 2025 Nov 19;20(11):e0336309. doi: 10.1371/journal.pone.0336309 (PMC12629489; doi:10.1371/journal.pone.0336309)
Supplement: S11 File — (DOCX) [file pone.0336309.s011.docx]

**S11 File. Relative percentages of Ergosterol content in absence and presence of Ag-*MO* and Ag-Zn-*MO*.**

| **Sample** | **E1** | **E2** | **E3** | **Mean** | **SD** |
| --- | --- | --- | --- | --- | --- |
| **Control** | 0.0063 | 0.0064 | 0.0062 | 0.0063 | 0.0001 |
| **Ag-MO** | 0.002 | 0.003 | 0.002 | 0.002 | 0.0005 |
| **Ag-Zn-MO** | 0.001 | 0.002 | 0.002 | 0.001 | 0.0005 |
