# Supplementary material for: Candidacidal effect of Moringa stabilized silver nanomaterials reveal disruption of cell wall integrity, efflux pump, vacuole homeostasis and virulence traits in Candida auris
Source: PLoS One. 2025 Nov 19;20(11):e0336309. doi: 10.1371/journal.pone.0336309 (PMC12629489; doi:10.1371/journal.pone.0336309)
Supplement: S12 File — (DOCX) [file pone.0336309.s012.docx]

**S12 File. Extracellular R6G concentrations for efflux pump mechanism of Ag-*MO***

| **Time** | **Positive Control** | **Ag-*MO*** | **Negative Control** |
| --- | --- | --- | --- |
| 0 min | 0.102 | 0.103 | 0.101 |
| 10 min | 0.184 | 0.178 | 0.104 |
| 20 min | 0.227 | 0.188 | 0.107 |
| 30 min | 0.229 | 0.198 | 0.106 |
| 40 min | 0.233 | 0.201 | 0.111 |
| 50 min | 0.236 | 0.217 | 0.122 |
| 60 min | 0.239 | 0.218 | 0.103 |
| 70 min | 0.246 | 0.224 | 0.123 |
| 80 min | 0.263 | 0.226 | 0.121 |
| 90 min | 0.274 | 0.232 | 0.124 |
