# Supplementary material for: Candidacidal effect of Moringa stabilized silver nanomaterials reveal disruption of cell wall integrity, efflux pump, vacuole homeostasis and virulence traits in Candida auris
Source: PLoS One. 2025 Nov 19;20(11):e0336309. doi: 10.1371/journal.pone.0336309 (PMC12629489; doi:10.1371/journal.pone.0336309)
Supplement: S13 File — (DOCX) [file pone.0336309.s013.docx]

**S13 File. Mode of inhibition via Lineweaver-Burk plot (Ag-*MO*)**

| **Time** | **1/s** | **1/v treated** | **1/v control** |
| --- | --- | --- | --- |
| 40 | 0.025 | 11.12 | 7.91 |
| 30 | 0.033333 | 11.71 | 7.33 |
| 20 | 0.05 | 11.89 | 8.41 |
| 10 | 0.1 | 14.92 | 9.77 |
