# Supplementary material for: Candidacidal effect of Moringa stabilized silver nanomaterials reveal disruption of cell wall integrity, efflux pump, vacuole homeostasis and virulence traits in Candida auris
Source: PLoS One. 2025 Nov 19;20(11):e0336309. doi: 10.1371/journal.pone.0336309 (PMC12629489; doi:10.1371/journal.pone.0336309)
Supplement: S14 File — (DOCX) [file pone.0336309.s014.docx]

**S14 File. Extracellular R6G concentrations for efflux pump mechanism of Ag-Zn-*MO***

| **Time** | **Positive Control** | **Ag-Zn-*MO*** | **Negative Control** |
| --- | --- | --- | --- |
| 0 min | 0.087 | 0.085 | 0.086 |
| 10 min | 0.112 | 0.097 | 0.086 |
| 20 min | 0.116 | 0.098 | 0.085 |
| 30 min | 0.119 | 0.101 | 0.084 |
| 40 min | 0.124 | 0.104 | 0.086 |
| 50 min | 0.13 | 0.109 | 0.085 |
| 60 min | 0.154 | 0.113 | 0.087 |
| 70 min | 0.201 | 0.135 | 0.089 |
| 80 min | 0.208 | 0.14 | 0.09 |
| 90 min | 0.214 | 0.143 | 0.091 |
