# Supplementary material for: Candidacidal effect of Moringa stabilized silver nanomaterials reveal disruption of cell wall integrity, efflux pump, vacuole homeostasis and virulence traits in Candida auris
Source: PLoS One. 2025 Nov 19;20(11):e0336309. doi: 10.1371/journal.pone.0336309 (PMC12629489; doi:10.1371/journal.pone.0336309)
Supplement: S15 File — (DOCX) [file pone.0336309.s015.docx]

**S15 File. Mode of inhibition via Lineweaver-Burk plot (Ag-Zn-*MO*)**

| **Time** | **1/s** | **1/v Treated** | **1/v Control** |
| --- | --- | --- | --- |
| 40 | 0.021 | 16.97 | 18.9 |
| 30 | 0.033 | 23.09 | 21.32 |
| 20 | 0.05 | 34.96 | 26.52 |
| 10 | 0.1 | 38.75 | 51.81 |
