# Supplementary material for: Candidacidal effect of Moringa stabilized silver nanomaterials reveal disruption of cell wall integrity, efflux pump, vacuole homeostasis and virulence traits in Candida auris
Source: PLoS One. 2025 Nov 19;20(11):e0336309. doi: 10.1371/journal.pone.0336309 (PMC12629489; doi:10.1371/journal.pone.0336309)
Supplement: S16 File — (DOCX) [file pone.0336309.s016.docx]

**S16 File. Toxicity of Ag-*MO* and Ag-Zn-*MO* on *C. elegans* showing number of *C. elegans* in the presence of Ag-*MO* and Ag-Zn-*MO* at sub-MIC concentration**

| **Day** | **Control**  **(number of *C.elegans*)** | **Ag-*MO***  **(number of *C.elegans*)** | **Ag-Zn-*MO***  **(number of *C.elegans*)** |
| --- | --- | --- | --- |
| 0.99 | 50 | 50 | 50 |
| 1 | 50 | 50 | 50 |
| 1.99 | 50 | 46 | 47 |
| 2 | 50 | 46 | 47 |
| 2.99 | 50 | 46 | 47 |
| 3 | 48 | 42 | 47 |
| 3.99 | 48 | 42 | 44 |
| 4 | 48 | 42 | 41 |
| 4.99 | 48 | 40 | 41 |
| 5 | 48 | 40 | 41 |
| 5.99 | 46 | 36 | 38 |
| 6 | 46 | 36 | 38 |
| 6.99 | 44 | 36 | 38 |
| 7 | 44 | 36 | 35 |
