# Supplementary material for: Candidacidal effect of Moringa stabilized silver nanomaterials reveal disruption of cell wall integrity, efflux pump, vacuole homeostasis and virulence traits in Candida auris
Source: PLoS One. 2025 Nov 19;20(11):e0336309. doi: 10.1371/journal.pone.0336309 (PMC12629489; doi:10.1371/journal.pone.0336309)
Supplement: S17 File — (DOCX) [file pone.0336309.s017.docx]

**S17 File. Percentage survival of *C. auris*–infected *C. elegans* in the presence of Ag-*MO* and Ag-Zn-*MO* until 7 days.**

| **Day** | **Control**  **(Number of *C.elegans*)** | **Ag-MO**  **(Number of *C.elegans*)** | **Ag-Zn-MO**  **(Number of *C.elegans*)** |
| --- | --- | --- | --- |
| 0.99 | 50 | 50 | 50 |
| 1 | 45 | 50 | 50 |
| 1.99 | 45 | 50 | 50 |
| 2 | 45 | 50 | 47 |
| 2.99 | 42 | 48 | 47 |
| 3 | 42 | 48 | 47 |
| 3.99 | 38 | 48 | 44 |
| 4 | 38 | 48 | 44 |
| 4.99 | 38 | 47 | 44 |
| 5 | 34 | 47 | 41 |
| 5.99 | 34 | 45 | 41 |
| 6 | 34 | 45 | 41 |
| 6.99 | 30 | 45 | 40 |
| 7 | 30 | 43 | 40 |
