# Supplementary material for: Candidacidal effect of Moringa stabilized silver nanomaterials reveal disruption of cell wall integrity, efflux pump, vacuole homeostasis and virulence traits in Candida auris
Source: PLoS One. 2025 Nov 19;20(11):e0336309. doi: 10.1371/journal.pone.0336309 (PMC12629489; doi:10.1371/journal.pone.0336309)
Supplement: S18 File — Bar graph with CFU log105/ ml on the y-axis after 24-h infection with control (-Ag-MO and - Ag-Zn-MO) and treated (+Ag-MO & + Ag-Zn-MO) C. auris depicting the killing of fungi by macrophages. (DOCX) [file pone.0336309.s018.docx]

**S18 File. Macrophage killing assay. Bar graph with CFU log10^5^/ ml on the y-axis after 24-h infection with control (-Ag-*MO* and - Ag-Zn-*MO*) and treated (+Ag-*MO* & +Ag-Zn-*MO) C. auris* depicting the killing of fungi by macrophages.**

| **Sample** | **(10^5^ CFU)**  **E1** | **(10^5^ CFU)**  **E2** | **(10^5^ CFU)**  **E3** | **Mean** | **Standard Deviation** |
| --- | --- | --- | --- | --- | --- |
| Control | 138.66 | 137.24 | 140.074 | 138.65 | 1.42 |
| Ag-MO | 46 | 44.58 | 47.41 | 45.99 | 1.41 |
| Ag-Zn-MO | 55.66 | 54.24 | 57.07 | 55.66 | 1.42 |
